# Revision of the termite family Rhinotermitidae (Isoptera) in New Guinea

**DOI:** 10.3897/zookeys.148.1826

**Published:** 2011-11-21

**Authors:** Thomas Bourguignon, Yves Roisin

**Affiliations:** 1 Evolutionary Biology and Ecology, CP 160/12, Université Libre de Bruxelles (ULB), Avenue F.D. Roosevelt 50, B-1050 Brussels, Belgium; 2 Present address: Graduate School of Environmental Science, Hokkaido University, Sapporo 060–0810, Japan

**Keywords:** termites, Papua New Guinea, Indonesia, new species

## Abstract

Recently, we completed a revision of the Termitidae from New Guinea and neighboring islands, recording a total of 45 species. Here, we revise a second family, the Rhinotermitidae, to progress towards a full picture of the termite diversity in New Guinea. Altogether, 6 genera and 15 species are recorded, among which two species, *Coptotermes gambrinus* and *Parrhinotermes barbatus*, are new to science. The genus *Heterotermes* is reported from New Guinea for the first time, with two species restricted to the southern part of the island. We also provide the first New Guinea records for six species of the genera *Coptotermes* and *Schedorhinotermes*. We briefly describe soldiers and imagoes of each species and provide a key based on soldier characters. Finally, we discuss the taxonomic and biogeographical implication of our results. A replacement name, *Schedolimulus minutides* Bourguignon, is proposed for the termitophilous staphylinid *Schedolimulus minutus* Bourguignon, to solve a question of secondary homonymy.

## Introduction

The Rhinotermitidae constitute one of the most widespread termite families, including numerous pest species. First established under the invalid name Mesotermitidae (Holmgren 1910a, b), then reestablished as Rhinotermitidae by Light (1921), the family originally included all termites possessing a fontanelle and a frontal gland in the imago and soldier castes, 8 malpighian tubules and hindgut with short anterior (pre-paunch) sections. Later on, the Stylotermitidae and Serritermitidae were separated as distinct families (Chatterjee and Thakur 1964, Emerson 1965), a position recently endorsed by Engel et al. (2009). The Rhinotermitidae now comprise several genera of uncertain affinities, such as *Prorhinotermes*, *Termitogeton* and *Psammotermes*, and two well-supported clades: (i) the Rhinotermitinae, including *Parrhinotermes*, *Schedorhinotermes* and the neotropical *Rhinotermes*-group; (ii) the Heterotermitinae + Coptotermitinae, comprising *Reticulitermes*, *Heterotermes* and *Coptotermes*. As presently defined, the Rhinotermitidae might still be paraphyletic with respect to the Serritermitidae or Termitidae, but the actual phylogeny of this group remains uncertain (Lo et al. 2004, Ohkuma et al. 2004, Inward et al. 2007).

The Rhinotermitidae are widely distributed across tropical, subtropical and temperate regions (Eggleton 2000). Whereas the genus *Prorhinotermes* is notable for its pantropical insular distribution (Emerson 1952), other genera are either pantropical (e.g. *Coptotermes*) or limited to one (e.g. *Termitogeton*) or a few zoogeographic areas (e.g. *Schedorhinotermes*). In southeast Asia, the Rhinotermitidae are represented by six genera: *Parrhinotermes*, *Schedorhinotermes*, *Coptotermes*, *Heterotermes*, *Prorhinotermes* and *Termitogeton*. All but the last one are also present in Australia. The former two belong to the subfamily Rhinotermitinae, whose soldiers are equipped to bite and smear a poisonous liquid with their elongated brush-like labrum (Quennedey and Deligne 1975). In the other genera, soldiers can simultaneously bite and emit a toxic or sticky chemical secretion from their frontal gland (Prestwich 1979, Šobotník et al. 2010a). Alates are also equipped with a frontal gland, variously developed according to the genus (Šobotník et al. 2010b).

Some recent revisions or compilations of distributional data are available for Rhinotermitidae in Southeast Asia and Australia. Faunal lists mention 21 species from Sundaland (Peninsular Malaysia to Borneo: Gathorne-Hardy 2004), 12 from Sulawesi (Gathorne-Hardy et al. 2000), 24 from Australia (Watson et al. 1998). However, prior to the beginning of our survey in 1978, only five species in two genera were known from New Guinea: *Coptotermes elisae* (Desneux, 1905), *Coptotermes obiratus* Hill, 1927, *Schedorhinotermes dimorphus* (Desneux, 1905), *Schedorhinotermes translucens* (Haviland, 1898), and *Schedorhinotermes celebensis* (Holmgren, 1911a). *Prorhinotermes inopinatus* Silvestri, 1909, was reported more recently (Gay and Barrett 1983). Findings of *Prorhinotermes* (identified as *Prorhinotermes inopinatus*), *Parrhinotermes* (as *Parrhinotermes browni* (Harris, 1958)) and *Termitogeton* (as *Termitogeton* nr. *planus* (Haviland, 1898)) were mentioned in studies focused on caste patterns (Roisin 1988a, b, Parmentier and Roisin 2003), bringing the total number of New Guinean species to 8 in 5 genera. The present revision is primarily based on extensive collections carried out in New Guinea between 1978 and 1995. We recorded 15 species in 6 genera, among which 2 species are new to science. The present work complements our series of monographic revisions on New Guinean Termitidae, in which we recorded a total of 45 species in 13 genera (Roisin 1990, Roisin and Pasteels 1996, 2000, Bourguignon et al. 2008).

## Materials and methods

### Biological material

Extensive termite collecting was carried out by J. M. Pasteels (in collection records: **JMP**), Y. Roisin (**YR**) and M. Leponce (**ML**) in New Guinea and some neighboring islands between 1978 and 1995 although records from Indonesian Papua are almost exclusively limited to the “bird’s neck” area, around Nabire and Kaimana (Figs 30, 82). Termite specimens were collected with tweezers and preserved in 80% alcohol or fixed in Bouin’s fluid or in a formol-alcohol-acetic acid (20:75:5) mixture. We also had the opportunity to examine samples collected in alcohol by Alfred E. and Eleanor Emerson in 1962–1963 (**AE**), as well as a few samples from other sources. Localities where specimens were collected are given as well as their approximate geographic coordinates (Appendix 1), obtained by cross-checking maps, Google^TM^ Earth positioning and the National Geospatial Agency GEOnet Names Server (http://geonames.nga.mil/ggmagaz/).

### Systematic characters

This study is based on the morphology of soldier and alate castes. Soldiers supply most of the important taxonomic information at the species level. The following characters are of major interest: size, general shape of the head, shape of mandibles, shape of postmentum, pilosity of head and number of antennal articles. Alates, when available, may give taxonomic information at the species level by the general shape of their head and pronotum.

### Measurements and their abbreviations

The measurements used, detailed below, follow the guidelines of Roonwal (1970).

**Figures 1–4.**
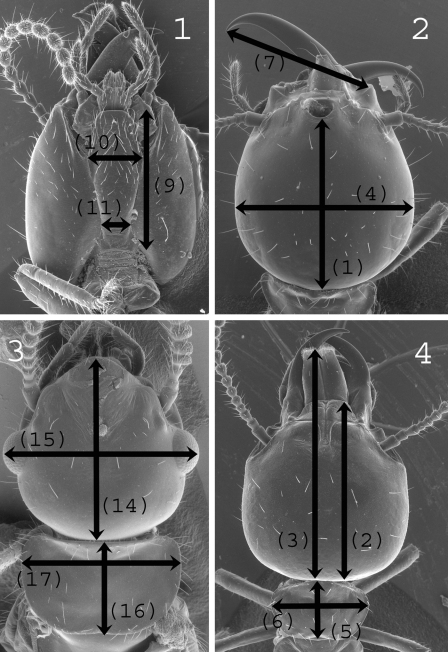
Measurements. **A-B, D** soldier: **(1)**, HLF, head length to fontanelle; **(2)**, HLC, head length to apex of the clypeus; **(3)**, HLL, head length to apex of the labrum; **(4)**, HW, head maximum width; **(5)**, PL, pronotum length; **(6)**, PW, pronotum width; **(7)**, RML, right mandible length; **(9)**, PML, postmentum length; **(10)**, MPW, maximum postmentum width; **(11)**, mPW, minimum postmentum width. **C** imago: **(14)**, HLC, head length to apex of the clypeus; **(15)**, HWE, head width with eyes; **(16)**, PL, pronotum length; **(17)**, PW, pronotum width.

**Soldiers.** (Fig. 1, 2, 4): **HLF** – Head length to fontanelle; **HLC** – Head length to apex of the clypeus; **HLL** – Head length to apex of the labrum; **HW**– Head maximum width; **PL** – Pronotum length; **PW** – Pronotum width; **RML** – Right mandible length; **LML** – Left mandible length; **PML** – Postmentum length; **MPW** –Maximum postmentum width; **mPW** – Minimum postmentum width; **T3L** – Hind tibia length.

**Imagoes.** (Fig. 3): **TBL** – Total body length (without wings); **HLC** – Head length to apex of the clypeus; **HWE** – Head width with eyes; **PL** – Pronotum length; **PW** – Pronotum width; **FWL** – Forewing length (with scale); **ED** – Eyes maximum diameter

### Microscopy

For scanning electron microscopy, specimens were dehydrated in a conventional ethanol series, impregnated for 24 h in hexamethyldisilazane, air dried and gold coated. Digital images were taken with a Philips XL 30 ESEM.

### Collections and their abbreviations

Species determinations were carried out after comparison with type series or identified specimens kept in the following museums

AMNHAmerican Museum of Natural History, New York, USA

BMNHNatural History Museum, London, UK

CUMZCambridge University Museum of Zoology, Cambridge, UK

IRSNBInstitut Royal des Sciences Naturelles, Brussels, Belgium

MVMAMuseum of Victoria, Abbotsford, Victoria, Australia

NHRSNaturhistoriska Riksmuseet, Stockholm, Sweden

Samples numbered #PNGT*** (Papua New Guinea Termites) or #IRJT*** (Irian Jaya Termites) are in the authors’ laboratory collection at the Université Libre de Bruxelles. The major part of this collection will ultimately be deposited at the IRSNB.

## Systematics

### Genus Coptotermes

Wasmann, 1896

http://species-id.net/wiki/Coptotermes

Termes (*Coptotermes*) Wasmann 1896: 629.

#### Type species.

*Termes gestroi* Wasmann, 1896, by monotypy.

#### Diagnosis.

Imago head circular, covered by many setae. Fontanelle indistinct and appearing as a small spot in the middle of the head. Large ocelli located above the eyes. Pronotum and abdomen densely hairy. Soldiers with pyriform head capsule, slightly hairy. Fontanelle very large, directed forward, through which the latex-like secretion of the frontal gland is discharged. Labrum short, triangular-shaped. Mandibles narrow and elongated, curved at tip; right mandible without subsidiary teeth and serrations; left one with a basal tooth and serrations. Antennae with 12 to 16 articles.

#### Distribution.

The genus *Coptotermes* is broadly distributed, occurring in all tropical and subtropical regions. It comprises species adapted to all major biomes, from tropical rainforest to arid steppes and deserts. Nests are generally found in logs, in the heartwood of living trees, or underground (Emerson 1971). Several species, such as *Coptotermes formosanus*, are major pests of buildings (Su and Scheffrahn 2000).

### Coptotermes elisae

(Desneux, 1905)

http://species-id.net/wiki/Coptotermes_elisae

Figs 5–9 30

Termes (*Coptotermes*) *Elisae*Desneux 1905: 368.
Coptotermes Elisae Desneux. Holmgren 1911a: 456.
Coptotermes hyaloapex Holmgren 1911a: 457 (synonymized by Gay 1963: 421–423).

#### Material examined.

**Syntypes:** **PAPUA NEW GUINEA:** **Morobe:** Simbang, 12.ix.1898 (L. Biró), alates only (IRSNB). **Other material.** **PAPUA NEW GUINEA: Madang:** Laing Island, 28.xi.1978 (JMP), with queen and alate (#PNGT22); Laing Island, 11.vi.1987 (YR), with alates (#PNGT1101); Laing Island, 24.viii.1988 (YR), nest within fallen *Erythrina* tree (#PNGT1260); Manam Island, 23.vi.1981 (JMP), feeding on live tree (#PNGT101); Bogia–Josephstaal road, 10 km S Guam bridge, 26.vi.1984 (YR) (#PNGT711); Hansa Point, 22.viii.1984 (YR) (#PNGT785): Awar, 16.ix.1984 (YR) (#PNGT831); Tabele (Manam Island), 19.ix.1984 (YR), in decaying palm (#PNGT839); Sepen No.1, 01.iii.1988 (YR) (#PNGT1164); Baitabag, v.1999 and 17.x.1999 (L. Čižek) (#7, #15, J. Šobotník’s collection). **Morobe:** Sirasira, 14.v.1988 (YR) (#PNGT1213); Bulolo, 22.v.1987 (YR), in dead hoop pine (*Araucaria cunninghammi*) trunk on the ground (#PNGT1080); **Central:** Brown River forest, 15.xi.1962 (AE), from dead branch on forest floor (AMNH); Brown River, 21.xi.1962 (AE), from standing dead stump in forest (AMNH); 24 km NE Port Moresby, 23.xi.1962 (AE), covered galleries over large surface of live tree, with hole to heart wood (AMNH); Subitana plantation, 24.xi.1962 (AE), from standing dead rubber tree in mature grove (AMNH); Sogeri, 15.vii.1984 (YR), in dead liana along tree trunk in small patch of forest (#PNGT740); Sogeri, 23.iii.1985 (JMP & YR) (#PNGT1005); **Oro:** Kokoda, 13.iii.1985 (JMP & YR) (#PNGT952); Kokoda, 17.iii.1985 (JMP & YR) (#PNGT981); Koiasi, 14.iii.1985 (JMP & YR) (#PNGT959); **New Ireland:** Konos, 29.v.1984 (JMP & YR) (#PNGT624); **Sandaun:** Yapsiei, 10.iii.1994 (YR & ML) (#PNGT1733 & 1734); **Fly:** Morehead, 25.iii.1989 (YR & ML) (#PNGT1447); Wipim, 01.iv.1989 (YR & ML) (#PNGT1509); Tabubil, 20.v.1990 (YR & ML) (#PNGT1541); Lake Murray, 24.v.1990 (YR & ML) (#PNGT1582); Lake Murray, 25.v.1990 (YR & ML), with queen (#PNGT1589); Nomad, 29.v.1990 (YR & ML) (#PNGT1609); Nomad, 31.v.1990 (YR & ML) (#PNGT1624); Nomad, 01.vi.1990 (YR & ML) (#PNGT1637); **Southern Highlands:** Pimaga, alt. 950m, 18.x.1988 (YR) (#PNGT1316); **INDONESIA:** **Papua:** Road Nabire-Mapia km 43, 26.xi.1995 (YR) (#IRJT171); Road Nabire-Mapia km 62, 18.xi.1995 (YR) (#IRJT72, 73); Coa, 22.xi.1995 (YR) (#IRJT123); Kaimana, 23.xi.1995 (YR) (#IRJT140).

**Figures 5–9.**
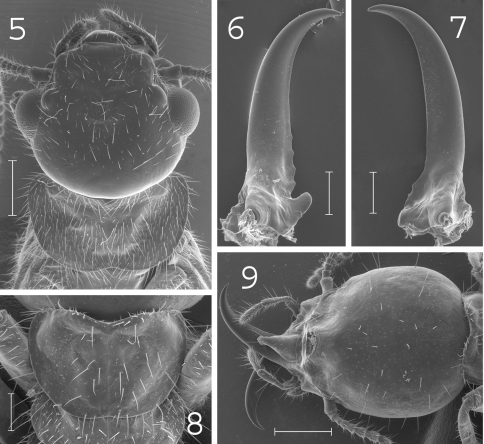
*Coptotermes elisae*. Imago: **5** head and pronotum. Soldier: **6** left mandible; **7** right mandible; **8** pronotum; **9** head. Scale bars: **5**, **9:** 0.5mm; **6**, **7**, **8:** 0.2mm.

#### Imago.

(Fig. 5).Head rounded and densely hairy. Large eyes. Pronotum wide and elongated, covered by numerous setae of medium and large size. Antennae with 20 to 22 articles. Measurements (mm) of 8 imagoes from the type colony and 4 imagoes from one other colony (between parentheses): TBL: 8.27–8.99 (7.81–8.12); HLC: 1.53–1.87 (1.48–1.62); HWE: 1.84–1.96 (1.79–1.81); PL: 1.04–1.12 (1.02–1.07) ; PW: 1.63–1.70 (1.59–1.61); FWL: n.a. (13–13.2); ED: 0.45–0.50 (0.45–0.5).

#### Soldier.

(Figs 6–9).Soldiers of large size. Head rounded, covered by about 40 setae. Fontanelle large, with opening well visible from above. Antennae generally with 16 articles. Pronotum elongated, covered by about 70 setae mainly located on the edges. Mesonotum, metanotum and abdomen bearing plenty of setae. Mandibles with tips extremely curved. Measurements (mm) of 99 soldiers from 33 colonies: HLF: 1.11–1.47; HLL: 1.70–2.20; HW: 1.21–1.54; PL: 0.38–0.64; PW: 0.77–1.03; RML: 0.97–1.20; MPW: 0.30–0.49; T3L: 1.05–1.34.

#### Comparisons.

*Coptotermes elisae* can be distinguished from other New Guinean species by its large rounded head with fontanelle opening well visible from above, its strongly curved mandibles and elongated pronotum in the soldier caste.

#### Distribution.

(Fig. 30).This species is widespread in New Guinean forests. It also occurs in New Ireland. It has also been reported from the following localities, but the relevant material was not examined: Bukaua (as *Coptotermes hyaloapex*: Holmgren 1911a), Popondetta (Gay 1963). According to Gathorne-Hardy (2004), it is present from Peninsular Malaysia throughout Sundaland.

#### Termitophiles.

*Coptophysa obesa* (Coleoptera: Staphylinidae) was found in colony #PNGT740 of this species in Sogeri (Roisin and Pasteels 1990).

### Coptotermes remotus

Hill, 1927

http://species-id.net/wiki/Coptotermes_remotus

Figs 10–15 31

Coptotermes remotus Hill 1927: 16.

#### Material examined.

**Syntypes:** **PAPUA NEW GUINEA: New Ireland:** Kavieng (as Kaewieng), 4.x.1923 (H.G. Wallace) (NMVA T-18703, T-18704). **Other material: PAPUA NEW GUINEA: Madang:** Hansa Point, 08.vii.1984 (YR) (#PNGT719); Awar, 11.ix.1984 (YR) (#PNGT828); Nubia, 17.ii.1988 (YR) (#PNGT1154); Potsdam plantation, 22.xi.1988 (YR) (#PNGT1333); Hatzfeldhafen, 25.viii.1984 (YR) (#PNGT795); Baitabag, 17.x.1999 (L. Čižek) (#1, J. Šobotník’s collection);**Sandaun:** Yapsiei, 10–11.iii.1994 (YR & ML) (#PNGT1735, 1744). **Central:** Sirinumu Dam, 7.iii.1985 (JMP & YR) (#PNGT923); **Fly:** Lake Murray, 23.v.1990 (YR & ML) (#PNGT1569). **INDONESIA:** **Papua:** Pusppenssat-IrJa, 13.xi.1995 (YR) (#IRJT6, 10, 12); Pusppenssat-IrJa, 19.xi.1995 (YR) (#IRJT84, 85); Pusppenssat-IrJa, 30.xi.1995 (YR) (#IRJT207); Road Nabire-Mapia km 43, 15.xi.1995 (YR) (#IRJT40, 41); Road Nabire-Mapia km 43, 26.xi.1995 (YR) (#IRJT169, 170); Road Nabire-Mapia km 62, 18.xi.1995 (YR) (#IRJT74, 75); Topo, 28.xi.1995 (YR) (#IRJT189); Sanoba, 29.xi.1995 (YR) (#IRJT195); Coa, 22.xi.1995 (YR) (#IRJT131): Kaimana, 23.xi.1995 (YR) (#IRJT141). **Samples included with doubt: PAPUA NEW GUINEA:** **East Sepik:** Koil Island, 17.vi.1981 (JMP) (#PNGT75); **Madang:** Hansa Point, 23.ix.1988 (YR) (#PNGT1281).

**Figures 10–15.**
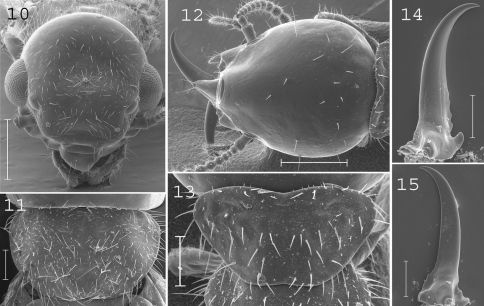
*Coptotermes remotus*. Imago: **10** head; **11** pronotum. Soldier: **12** head; **13** pronotum; **14** left mandible; **15** right mandible. Scale bars: **10**, **12:** 0.5mm; **11**, **13**, **14**, **15:** 0.2mm.

#### Imago.

(Figs 10–11).Head densely hairy. Pronotum with many medium and large setae. Antennae with 17 articles. Measurements (mm) of 6 imagoes from 1 colony: TBL: 5.75–6.39; HLC: 1.09–1.18; HWE: 1.30–1.38; PL: 0.57–0.73; PW: 0.97–1.08; FWL: 9.92–11.09; ED: 0.33–0.44.

#### Soldier.

(Figs 12–15). Soldiers of small size. Head slightly longer than broad, covered by about 20 setae. Fontanelle with opening directed forward, not visible from above. Antennae generally with 14 articles, but occasionally with only 13 articles. Pronotum larger anteriorly than posteriorly, trapezoid-shaped, covered by about 50 setae. Mandibles with tips extremely curved. Right mandible with four serrations. Measurements (mm) of 5 soldiers from the type colony and 72 soldiers from 24 colonies (parentheses): HLF: 1.11–1.19 (1.01–1.29); HLL: 1.45–1.56 (1.23–1.63); HW: 1.00–1.04 (0.83–1.11); PL: 0.36–0.39 (0.29–0.46); PW: 0.67–0.70 (0.59–0.78); RML: 0.78–0.83 (0.69–0.85); MPW: 0.28–0.31 (0.26–0.38); T3L: 0.83–0.89 (0.68–0.99).

#### Comparisons.

This species shows variation in size along its distribution range although no consistent characters allowed us to split it up. *Coptotermes remotus* most resembles the Australian species *Coptotermes lacteus* and the Malayan ones *Coptotermes bentongensis* and *Coptotermes sepangensis*. These last two species occur in sympatry and are morphologically undistinguishable, making them probable synonyms. Soldiers of *Coptotermes remotus* can be distinguished from other New Guinean species by their small size and mandibles curved at the tip.

#### Distribution.

This species, originally described from Kavieng, New Ireland, is widespread throughout New Guinean forests. It is abundant in the bird’s neck area of Indonesian Papua, but only a few specimens were collected from Papua New Guinea (Fig. 31).

#### Termitophiles.

*Coptophysella pulposa* (Coleoptera: Staphylinidae) was found in colony #PNGT795 of this species in Hatzfeldthafen (Roisin and Pasteels 1990). A possibly new species of *Coptophysella* was also found in colony #IRJT12 in Pusppenssat–IrJa (new record).

### Coptotermes grandiceps

Snyder, 1925

http://species-id.net/wiki/Coptotermes_grandiceps

Figs 16–21 30

Coptotermes grandiceps Snyder 1925: 401.
Coptotermes solomonensis Snyder 1925: 403 (synonymised by Hill 1942: 153).
Coptotermes obiratus Hill 1927: 17, **new synonymy.**
Coptotermes solomonensis Hill 1927: 19 (junior primary homonym of *Coptotermes solomonensis* Snyder; synonymised by Hill 1942: 153).
Coptotermes froggatti Light and Davis 1929: 62 (synonymised by Hill 1942: 153).

#### Material examined.

**Topotype:** **SOLOMON ISLANDS:** Tulaghi (as Tulagi), iii.1933 (R.A. Lever) (AMNH). **Holotype of *Coptotermes obiratus*:PAPUA NEW GUINEA: Central:** Waima, vii.1922 (G.F. Hill) (NMVA).

#### Other materials.

**PAPUA NEW GUINEA: Oro:** Mambare River, 27.iv.1922 (G.F. Hill) (NMVA). **Fly:** Morehead, 25.iii.1989 (YR & ML) (#PNGT1447); Wipim, 30.iii.1989 (YR & ML) (#PNGT1488); Lake Murray, 22.v.1990 (YR & ML) (#PNGT1561); Lake Murray, 25.v.1990 (YR & ML) (#PNGT1594); Nomad, 31.v.1990 (YR & ML) (#PNGT1627); Nomad, 02.vi.1990 (YR & ML) (#PNGT1651); **Central:** Subitana plantation, 24.xi.1962 (AE), in stump of rubber tree (AMNH); Sirinumu Dam, 06.iii.1985 (JMP & YR) (#PNGT916); Sirinumu Dam, 08.iii.1985 (JMP & YR) (#PNGT929); Varirata National Park, 06.xii.1988 (YR & Phille P. Daur) (#PNGT1346); **National Capital:** 2 km E Port Moresby,18.xi.1962 (AE), from log in dry eucalypt savanna (AMNH); 8 km E Port Moresby, 16.xi.1962 (AE), under log in eucalypt savanna (AMNH); 8 km E Port Moresby, 18.xi.1962 (AE), from dead log in ravine woods in savanna (AMNH); 10 km NW Port Moresby, 20.xi.1962 (AE), from log on ground in eucalypt savanna (AMNH); 19 km NW Port Moresby, 22.xi.1962 (AE), from fallen log in savanna (AMNH); **INDONESIA: Papua:** Pusppenssat-IrJa, 13.xi.1995 (YR) (#IRJT17).

**Figures 16–21.**
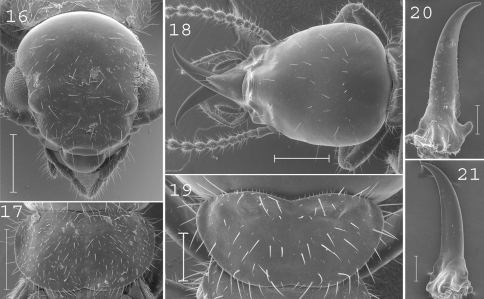
*Coptotermes grandiceps*. Imago: **16** head; **17** pronotum. Soldier: **18** head; **19** pronotum; **20** left mandible; **21** right mandible. Scale bars: **16**, **17**, **18:** 0.5mm; **19**, **20**, **21:** 0.2mm.

#### New synonymy.

In his revision of termites from Australia, Hill (1942) mentioned that soldiers of *Coptotermes grandiceps* and *Coptotermes obiratus* are morphologically indistinguishable, the two species being potential synonyms. After examining material of the two species, we reached the same conclusion and therefore consider *Coptotermes obiratus* as a junior synonym of *Coptotermes grandiceps*.

#### Imago.

(Figs 16–17).Head moderately hairy. Pronotum covered by many setae of medium size. Antennae with 20 articles. Measurements (mm) of 6 imagoes from one colony: TBL: 6.08–7.55; HLC: 1.19–1.27; HWE: 1.33–1.41; PL: 0.73–0.82; PW: 1.23–1.29; FWL: 9.97–10.61; ED: 0.31–0.42.

#### Soldier.

(Figs 18–21). Soldiers of large size. Head elongated, larger posteriorly than anteriorly, covered by about 30 setae. Fontanelle narrow, with opening directed frontally, not visible from above. Antennae generally with 15 articles, sometimes with 14 articles. Pronotum wide, moderately long, covered by about 60 setae. Mandibles elongated, curved at tip. Measurements (mm) of holotype of *Coptotermes obiratus*, 1 soldier from the type colony of *Coptotermes grandiceps* [brackets], and27 soldiers from 9 colonies (parentheses): HLF: 1.53 [1.52] (1.21–1.44); HLL: 2.17 [2.19] (1.89–2.20); HW: 1.38 [1.31] (1.17–1.33); PL: 0.51 [0.50] (0.40–0.56); PW: 0.97 [0.95] (0.79–1.00); RML: 1.17 [1.24] (1.03–1.15); MPW: 0.48 [0.42] (0.31–0.46); T3L: 1.22 [n.a.] (1.07–1.21).

#### Comparisons.

This species is closely allied to *Coptotermes pamuae* from which it can be distinguished by its larger soldiers with more curved mandibles.

#### Distribution.

(Fig. 30). *Coptotermes grandiceps*, originally described from the Solomon Islands, occurs in southern New Guinea and the Papuan peninsula.

### Coptotermes pamuae

Snyder, 1925

http://species-id.net/wiki/Coptotermes_pamuae

Figs 22–25 31

Coptotermes pamuae Snyder, 1925: 402.

#### Material examined.

**Paratype soldier from type colony:** **SOLOMON ISLANDS:** **San Cristobal:** Pamua, viii.1916 (W.M. Mann) (AMNH). **PAPUA NEW GUINEA:** **National Capital:** UPNG campus, 04.xii.1988 (YR) (#PNGT1338); **Central:** Varirata National Park, 06.xii.1988 (YR & Phille P. Daur) (#PNGT1348); **Fly:** Morehead, 27.iii.1989 (YR & ML) (#PNGT1466); Wipim, 30.iii.1989 (YR & ML) (#PNGT1486).

#### Imago.

Unknown.

#### Soldier.

**Figures 22–25.**
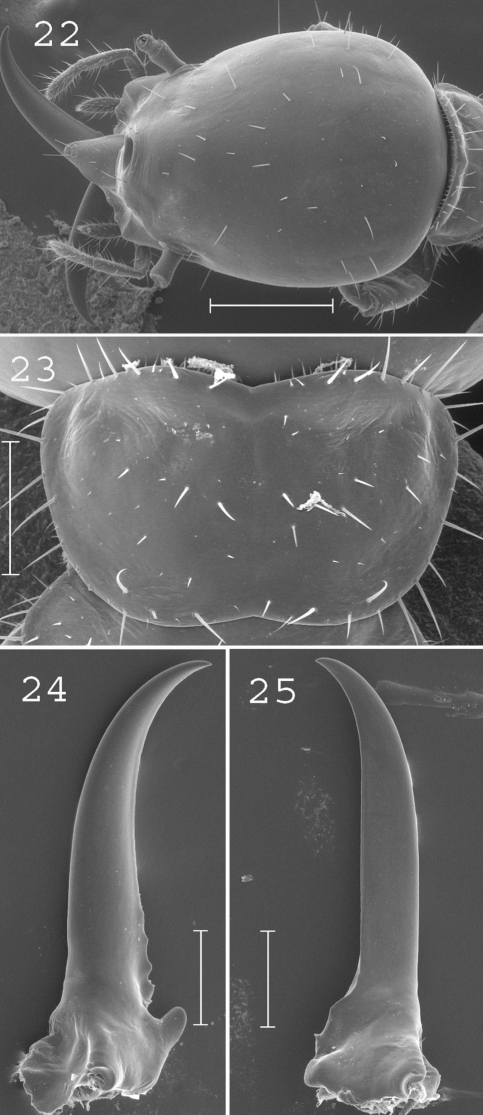
*Coptotermes pamuae*. Soldier: **22** head; **23** pronotum; **24** left mandible; **25** right mandible. Scale bars: **2:2** 0.5mm; **23**, **24**, **25:** 0.2mm.

(Figs 22–25). Soldiers of medium size. Head elongated, egg-shaped, slightly larger posteriorly than anteriorly, covered by about 30 setae. Fontanelle narrow, with opening directed forward. Antennae generally with 13 articles, sometimes with 14 articles. Pronotum short and narrow, covered by about 40 setae. Mandibles elongated, slightly curved at tip. Measurements (mm) of 1 paratype and 12 soldiers from 4 colonies (parentheses): HLF: 1.28 (1.11–1.27); HLL: 1.70 (1.58–1.79); HW: 1.12 (1.00–1.10); PL: 0.41 (0.31–0.44); PW: 0.79 (0.63–0.74); RML: 0.77 (0.89–0.98); MPW: 0.37 (0.28–0.32); T3L: 0.82 (0.92–1.08).

#### Comparisons.

This species is closely related and imperfectly separated from *Coptotermes grandiceps*. However, the size and morphological differences between the two species are high enough to retain them as distinct taxa, even though some individuals cannot be unambiguously assigned. The same was already observed in the related Australian species *Coptotermes acinaciformis* Froggatt, which is believed to form a species complex (Brown et al. 1990). The main criteria differentiating *Coptotermes pamuae* from *Coptotermes grandiceps* are its smaller soldiers with less hairy pronotum and less curved mandibles.

#### Distribution.

(Fig. 31). This species, originally described from the Solomon Islands, was only collected in southern Papua New Guinea (Fly savannas and the Port Moresby region).

### Coptotermes gambrinus sp. n.

urn:lsid:zoobank.org:act:8E27AAD1-29A7-471B-BD03-58F21AC53514

http://species-id.net/wiki/Coptotermes_gambrinus

Figs 26–29 31

#### Holotype.

**Soldier: PAPUA NEW GUINEA: Morobe:** Bulolo, 22.v.1987 (YR), from stump of klinkii pine (*Araucaria hunsteinii*) (#PNGT1077). **Paratypes: PAPUA NEW GUINEA: Morobe:** Soldiers and workers from same colony as holotype, same data (#PNGT1077); Mount Susu, 19.v.1988 (YR), in dead branch of klinkii pine on the ground (#PNGT1232); McAdam National Park, 20.v.1988 (YR) (#PNGT1238). **Madang:** Hatzfeldhafen, 23.ix.1984 (YR) (#PNGT847); **Oro:** Kokoda, 13.iii.1985 (JMP & YR) (#PNGT950); Popondetta, 18.iii.1985 (JMP & YR) (#PNGT986). **Central:** Sirinumu dam, 07.iii.1985 (JMP & YR) (#PNGT918); Brown River, 21.iii.1985 (JMP & YR) (#PNGT996); **East New Britain:** Warengoi, 19.v.1984 (JMP & YR) (#PNGT562, 567).

#### Imago.

unknown.

#### Soldier.

**Figures 26–29.**
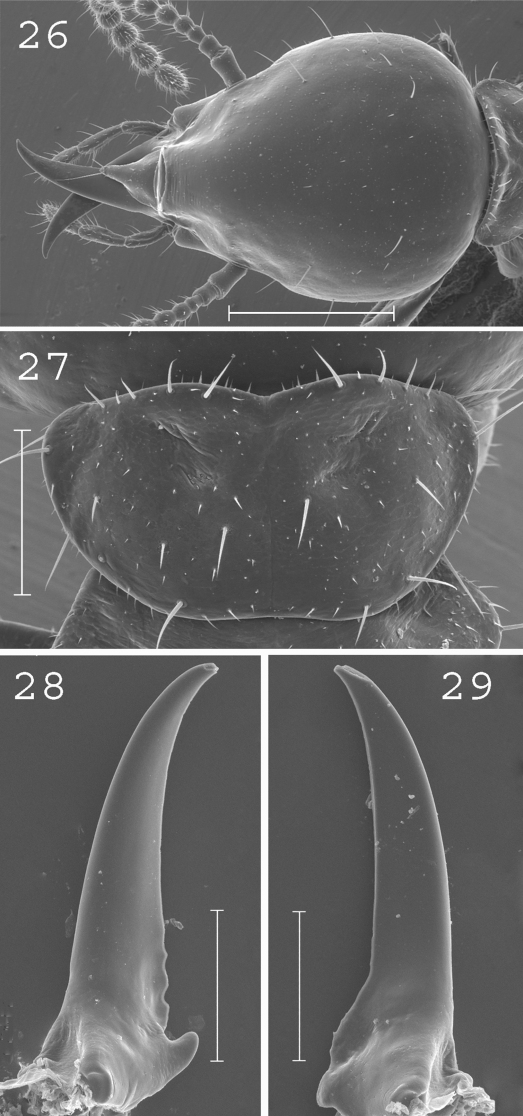
*Coptotermes gambrinus*. Soldier: **26** head; **27** pronotum; **28** left mandible; **29** right mandible. Scale bars: **26:** 0.5mm **27**, **28**, **29:** 0.2mm.

(Figs 26–29). Soldiers of small size. Head twice broader posteriorly than anteriorly (at the level of mandibles), covered by about 10 setae. Antennae generally with 15 articles. Pronotum covered by about 15 setae, slightly larger anteriorly than posteriorly. Mandibles very short, with apex almost not curved. Measurements (mm) of 30 soldiers from 10 colonies: HLF: 0.92–1.07; HLL: 1.22–1.48; HW: 0.82–0.98; PL: 0.27–0.41; PW: 0.50–0.70; RML: 0.56–0.74; MPW: 0.23–0.36; T3L: 0.75–0.88.

**Figure 30.**
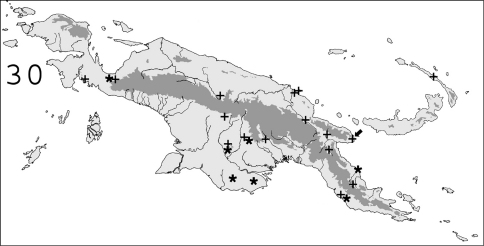
Known distribution in New Guinea of: ✚ *Coptotermes elisae*; ∗ *Coptotermes grandiceps*. Arrow points to type locality.

**Figure 31.**
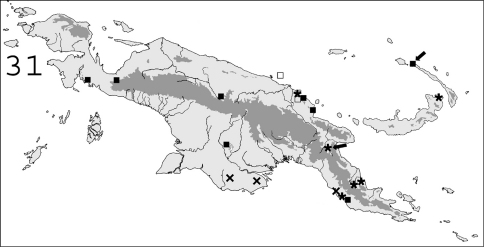
Known distribution in New Guinea of: ∗ *Coptotermes gambrinus*; ✖ *Coptotermes pamuae*; ■ *Coptotermes remotus*; □ doubtful samples of *Coptotermes remotus*. Arrows point to type localities.

#### Comparisons.

This species is allied to *Coptotermes remotus* from which it is easily recognisable by its shorter and less curved soldier mandible tips.

#### Distribution.

(Fig. 31). This species occurs in Eastern New Guinea and in New Britain.

#### Etymology.

We named this species in honor of *Gambrinus*, a legendary character from Flanders, famous for enjoying life.

### Genus Heterotermes

Froggatt, 1897

http://species-id.net/wiki/Genus_Heterotermes

Heterotermes Froggatt 1897: 518.

#### Type species.

*Heterotermes platycephalus* Froggatt, 1897, by monotypy.

#### Diagnosis.

Imago head roughly oval, narrower anteriorly than posteriorly. Fontanelle small, located in the middle of the head. Eyes small and flat. Ocelli situated in front of the head, before eyes. Antennae generally with 15 to 19 articles. Pronotum elongated, narrower than head. Soldier head long and narrow, rectangular-shaped. Fontanelle small, circular, situated forward. Labrum short to medium-sized, about half as long as mandibles. Mandibles sabre-shaped, slightly curved at tips. Left mandible with a tooth and some serrations at the base. Right mandible without basal tooth and serrations. Antennae with 13 to 18 articles.

#### Distribution.

Most species of *Heterotermes* are tropical (Emerson 1971). This genus was known from the Neotropics, northern Africa, Asia (from the Arabic peninsula to Indonesia), and Australia, occurring from humid forests to desert edges. Here, we extend its known distribution to southern New Guinea.

### Heterotermes vagus

(Hill, 1927)

http://species-id.net/wiki/Heterotermes_vagus

Figs 32–36 44

Leucotermes vagus Hill 1927: 53–55.
Heterotermes vagus (Hill). Hill 1942: 134–136.
Leucotermes venustus Hill 1927: 55. **New synonymy.**
Heterotermes venustus (Hill). Hill 1942: 131–134.

#### Material examined.

**Lectotype and paralectotype soldier: AUSTRALIA:** **Northern Territory:** Darwin, 01.viii.1914 (G.F. Hill) (NMVA #T-10848, #T-18705) **Lectotype of *Heterotermes venustus*:** **AUSTRALIA:** **Northern Territory:** Stapleton, 4.xi.1914 (G.F. Hill) (NMVA #T-10850). **Other material:** **PAPUA NEW GUINEA:** **Fly:** Morehead, 23.iii.1989 (YR & ML) (#PNGT1419, 1420, 1422); Morehead, 25.iii.1989 (YR & ML) (#PNGT1441); Lake Murray, 25.v.1990 (YR & ML) (#PNGT1588, 1597).

#### New synonymy.

Hill (1942) pointed out the similarity of *Heterotermes venustus* and *Heterotermes vagus*, but maintained both names arguing that *Heterotermes venustus* has a larger labrum and antennae with more articles. However, after comparing the type series of both species, we did not notice any difference in these characters, nor in any other morphological feature. For this reason, we consider these two species as synonyms and hereby give precedence to *Heterotermes vagus*.

**Figures 32–36.**
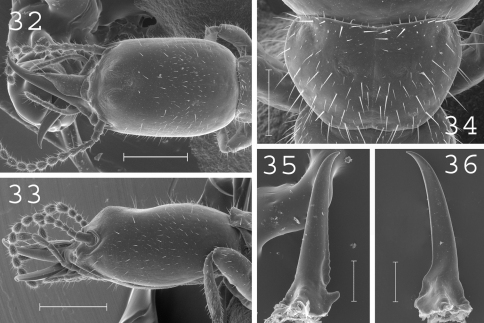
*Heterotermes vagus*. Soldier: **32** head in dorsal view; **33** head in lateral view; **34** pronotum; **35** left mandible; **36** right mandible. Scale bars: **32**, **33:** 0.5mm; **34**, **35**, **36:** 0.2mm.

#### Imago.

Unknown from New Guinea (see Hill 1942 for further details).

#### Soldier.

(Figs 32–36). Soldiers of small size. Head elongated, with a prominent hump at front, covered by plenty of short setae. Labrum elongated with sharp tip. Antennae generally with 13 articles. Pronotum short and narrow, covered by about 50 setae. Postmentum slightly narrow, without setae. Mandibles slightly curved at tips. Measurements (mm) of lectotype of *Heterotermes vagus*, lectotype of *Heterotermes venustus* [brackets] and 18 soldiers from 6 colonies (parentheses): HLC: 1.32 [1.43] (1.12–1.37); HLL: 1.75 [1.83] (1.48–1.76); HW: 0.80 [0.82] (0.74–0.83); PL: 0.40 [0.41] (0.32–0.50); PW: 0.55 [0.62] (0.49–0.60); RML: 0.92 [0.89] (0.80–0.93); MPW: 0.34 [0.31] (0.29–0.33); T3L: 0.65 [0.66] (0.54–0.66).

#### Distribution.

(Fig. 44).This species was collected in Sourthern Papua New Guinea. It is also known from northernmost Queensland and Northern Territory (Australia) (Watson and Abbey 1993).

### Heterotermes paradoxus

(Froggatt, 1898)

http://species-id.net/wiki/Heterotermes_paradoxus

Figs 37 44

Termes paradoxus Froggatt, 1898: 728.
Heterotermes paradoxus (Froggatt). Hill 1932: 146.

#### Material examined.

**AUSTRALIA: Northern Territory:** 37 km SE Newcastle Waters, 16.vi.1936 (coll. det. G.F. Hill) (ANIC #10–2186); **Queensland:** Weipa mission, 05.x.1957 (F.J. Gay & J.H. Calaby) (ANIC #10–8659); **PAPUA NEW GUINEA: Central:** Sogeri, 14.vii.1984 (YR) (#PNGT731); Sogeri, 4.ii.1985 (YR) (#PNGT855); Sirinumu Dam, 6.iii.1985 (JMP & YR) (#PNGT912); **Fly:** Morehead, 24.iii.1989 (YR & ML) (#PNGT1431); Wipim, 29.iii.1989 (YR & ML) (#PNGT1471); Wipim, 2.iv.1989 (YR & ML) (#PNGT1517); Lake Murray, 22.v.1990 (YR & ML) (#PNGT1562); Lake Murray, 24.v.1990 (YR & ML) (#PNGT1577); Lake Murray, 25.v.1990 (YR & ML) (#PNGT1590a,); Lake Murray, 27.v.1990 (YR & ML) (#PNGT1598, 1606). **INDONESIA: Papua:** Kaimana, 21.xi.1995 (YR) (#IRJT104, 111, 112); Kaimana, 23.xi.1995 (YR) (#IRJT142), two samples with alates (#IRJT143, 144); Pusppenssat-IrJa, 26.xi.1995 (YR) (#IRJT166).

**Figures 37–43.**
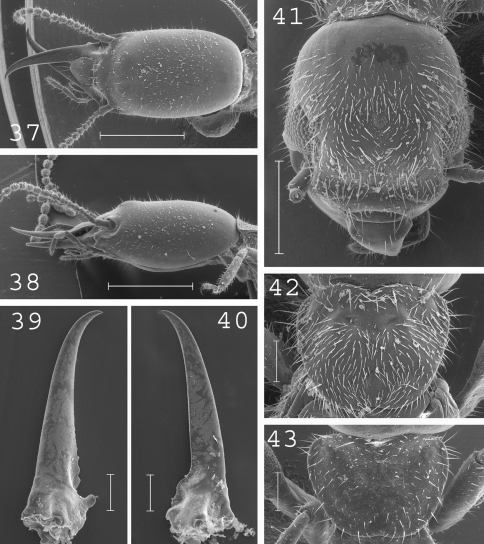
*Heterotermes paradoxus*. Soldier: **37** head in dorsal view; **38** head in lateral view; **39** left mandible; **40** right mandible; **43** pronotum. Imago: **41** head; **42** pronotum. Scale bars: **37**, **38:** 1mm; **41:** 0.5mm; **39**, **40**, **42**, **43:** 0.2mm.

**Figure 44.**
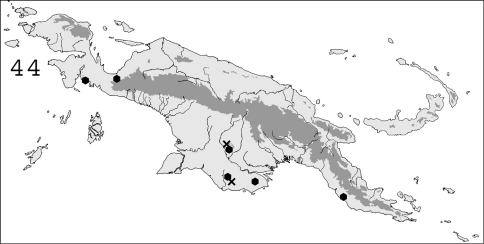
Known distribution in New Guinea of: ✖ *Heterotermes vagus*; ● *Heterotermes paradoxus*.

#### Imago.

(Figs 41–42).Head slightly elongated covered by several hundreds of setae. Pronotum elongated and moderately wide, covered by several hundreds of setae. Antennae generally with 18 articles, rarely with 17 articles. Eyes flat. Small ocelli in front of the eyes. Measurements (mm) of 12 imagoes from 1 colony: TBL: 5.16–6.31; HLC: 1.13–1.29; HWE: 0.98–1.14; PL: 0.59–0.64; PW: 0.76–0.85; FWL: 8.79–9.92; ED: 0.25–0.33.

#### Soldier.

(Figs 37–40, 43) Soldiers of large size. Head elongated, slightly rounded in the corners, with a well developed hump at front. Labrum with rounded apex. Antennae with 16 or 17 articles. Pronotum elongated, covered by numerous setae. Mandibles elongated, sligthly curved at tip. Measurements (mm) of 55 soldiers from 19 colonies: HLC: 1.34–1.98; HLL: 1.63–2.40; HW: 0.86–1.16; PL: 0.39–0.60; PW: 0.62–0.87; RML: 1.03–1.34; MPW: 0.35–0.48; T3L: 0.70–0.93.

#### Comparisons.

Morphological variation occurs along the distribution range of *Heterotermes paradoxus*, though it appears insufficient to recognize distinct species. *Heterotermes paradoxus* differs from *Heterotermes vagus* by the larger size of its soldiers, its less elongated labrum with rounded end and its antennae with 16 or 17 articles.

#### Distribution.

(Fig. 44).This species is widespread in southern Papua New Guinea, and was found on both coasts of the “bird’s neck” in Indonesian Papua. It is also known from northern Australia, especially Queensland (Watson & Abbey 1993). It occurs in savanna as well as in forest.

### Genus Parrhinotermes

Holmgren, 1910

http://species-id.net/wiki/Parrhinotermes

Parrhinotermes Holmgren 1910a: 285.

#### Type species.

*Termes aequalis* Haviland, 1898, by monotypy (as *Termes aqualis*, incorrect spelling).

#### Diagnosis.

Imago head approximately circular, with fontanelle situated between eyes. Frons with a slightly visible groove. Labrum short, inclined downward, without groove. Antennae with 16 or 17 articles. Pronotum relatively short, generally of the same width or slightly narrower than head. Soldier head rectangular to ovoid. Frons and clypeus with a groove, from the narrow fontanelle to the beginning of the labrum. Labrum elongated, crossed by a groove in the middle, and garnished with an apical brush. Base of mandibles serrated. Left mandible with two subsidiary teeth. Right mandible with one subsidiary tooth. Antennae with 13 articles.

#### Distribution.

This genus is known from the Oriental region, northeastern India, the Papuan region and northern Australia (in northern Queensland) (Emerson 1955, Tho 1992). It occurs in tropical rainforest where it nests in dead logs.

### Parrhinotermes browni

(Harris, 1958)

http://species-id.net/wiki/Parrhinotermes_browni

Figs 45–49 55

Schedorhinotermes browni Harris 1958: 59.
Parrhinotermes browni (Harris): Roisin 1988a: 22.

#### Material examined.

**Paratypes, soldiers and workers:** **SOLOMON ISLANDS: Guadalcanal:** Gold Ridge, 22.iii.1955 (E.S. Brown) (Brit. Mus. 1957–137, BMNH). **Other material: PAPUA NEW GUINEA:** **East Sepik:** Tsenap, 18.v.1929 (K.P. Schmidt), from log on ground (AMNH); **Madang:** Yagaum Hospital, 07.iv.1983 (YR) (#PNGT303, 306); Yagaum Hospital, 13.v.1983 (YR) (#PNGT340, 341); Bunapae, 18.ii.1984 (YR) (#PNGT546); Bunapae, 12.vi.1984 (YR) (#PNGT681); Guam bridge, 09.viii.1984 (YR), with royal pair (#PNGT782); Guam bridge, 12.ii.1985 (JMP & YR), with royal pair (#PNGT867); Guam bridge, 9.vi.1986 (YR), large colony with royal pair in rotten wood (#PNGT1023), and small sample (#PNGT1024); Sepen No.1, 29.vi.1986 (YR) (#PNGT1031); Hatzfeldthafen, 22.viii.1987 (YR), with primary king and ergatoid queen (#PNGT1130); Braham mission, 06.v.1988 (YR) (#PNGT1204); Wanuma, 05.viii.1969 (R. Zweifel) (AMNH); **Morobe:** Busu River, 17.xii.1962 (AE) (AMNH); 18 mi W Lae, 28.xi.1962 (AE), nest with royal pair in large log on forest floor (AMNH); Bulolo, 22.v.1987 (YR), in hoop pine (*Araucaria cunninghamii*) plantation (#PNGT1081); McAdam National Park, 20.v.1988 (YR) (#PNGT1236); **Sandaun:** Yapsiei, 11.iii.1994 (YR & ML) (#PNGT1742); Yapsiei, 12.iii.1994 (YR & ML), in standing dead wood, with alates (#PNGT1751); **Central:** Subitana plantation, xi.1962 (AE) (AMNH). **INDONESIA: Papua:** Pusppenssat-IrJa, 13.xi.1995 (YR) (#IRJT7, 9); Pusppenssat-IrJa, 14.xi.1995 (YR) (#IRJT28); Pusppenssat-IrJa, 15.xi.1995 (YR) (#IRJT39); Pusppenssat-IrJa, 29.xi.1995 (YR) (#IRJT203); road Nabire-Mapia km 43, 26.xi.1995 (YR) (#IRJT167).

**Figures 45–49.**
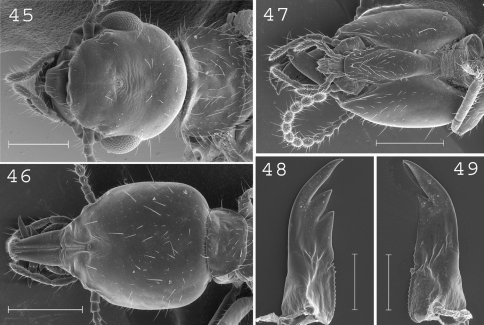
*Parrhinotermes browni*. Imago: **45** head. Soldier: **46** head in dorsal view; **47** head in ventral view; **48** left mandible; **49** right mandible. Scale bars: **45**, **46**, **47:** 0.5mm **48**, **49:** 0.2mm.

**Figures 50–54.**
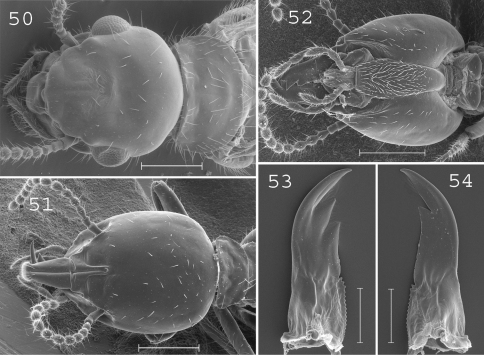
*Parrhinotermes barbatus*. Imago: **50** head. Soldier: **51** head in dorsal view; **52** head in ventral view; **53** left mandible; **54** right mandible. Scale bars: **50**, **51**, **52:** 0.5mm; **53**, **54:** 0.2mm.

**Figure 55.**
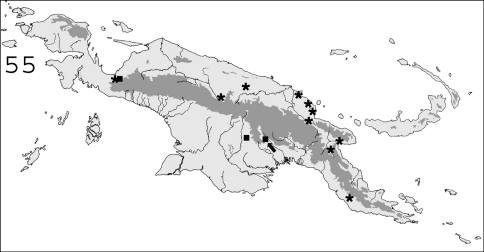
Known distribution in New Guinea of: ∗ *Parrhinotermes browni*; ■ *Parrhinotermes barbatus*. Arrow points to type locality.

#### Imago.

(Fig. 45). Head covered by about 15 setae with posterior edges strongly rounded, frons and clypeus short. Pronotum covered by about 100 setae. Antennae with 17 articles. Measurements (mm) of 6 imagoes from 1 colony: TBL: 5.76–6.34; HLC: 1.04–1.18; HWE: 1.19–1.26; PL: 0.47–0.65; PW: 0.90–0.95; FWL: 8.34–8.93; ED: 0.27–0.36.

#### Soldier.

(Figs 46–49) Head rectangular-shaped with edges slightly rounded, covered by about 50 small setae and 10 large ones. Labrum elongated, reaching the tip of mandibles, with a rounded apical brush. Postmentum covered by setae only on its upper part. Antennae with 13 articles. Mandibles straight, slightly curved at tip, slightly serrated at base. Measurements (mm) of 6 soldiers of the type colony and 39 soldiers from 13 colonies (parentheses): HLC: 1.13–1.19 (1.01–1.33); HLL: 1.56–1.64 (1.40–1.79); HW: 0.90–0.94 (0.86–1.05); PW: 0.52–0.56 (0.51–0.68); RML: 0.69–0.73 (0.64–0.78); MPW: 0.27–0.31 (0.27–0.34); mPW: 0.16–0.18 (0.16–0.22); T3L: 0.75–0.81 (0.70–0.89).

#### Distribution.

(Fig. 55). This species, originally described from the Solomon Islands, is common in Northern New Guinea and in the bird’s neck area.

#### Termitophiles.

Several species of Trichopseniini (Coleptera, Staphylinidae) were reported from this species (Bourguignon et al. 2007): *Parrhinopsenius brevipilosus*, *Parrhinopsenius longipilosus*, *Parrhinopsenius hirsutus*, *Parrhinopsenius parvus*, and one specimen of *Schedolimulus latus*.

### Parrhinotermes barbatus sp. n.

urn:lsid:zoobank.org:act:10EADEB0-140F-46D1-BEED-EFD6E6FA8C01

http://species-id.net/wiki/Parrhinotermes_barbatus

Figs 50 55

#### Holotype.

**Soldier: PAPUA NEW GUINEA: Southern Highlands:** Lake Kutubu, 11.x.1988 (YR) (#PNGT1285). **Paratypes: PAPUA NEW GUINEA: Southern Highlands:** Lake Kutubu, 11.x.1988 (YR), two colonies, one with alates, the other one with late nymphs (#PNGT1285, 1286); Lake Kutubu, 12.x.1988 (YR), with late nymphs (#PNGT1292); Lake Kutubu, 13.x.1988 (YR), with late nymphs (#PNGT1296); Pimaga, 16.x.1988 (YR) (#PNGT1306); Pimaga, 17.x.1988 (YR), with late nymphs (#PNGT1315); **Fly:** Nomad, 31.v.1990 (YR & ML), two colonies in dead wood, with queen (#PNGT1628, 1630); Nomad, 31.v.1990 (YR & ML) (#PNGT1650). **INDONESIA: Papua:** Road Nabire-Mapia km 62, 18.xi.1995 (YR) (#IRJT71).

#### Imago.

(Fig. 50). Head covered by about 15 setae with posterior margin strongly rounded, frons and clypeus of medium size. Pronotum covered by about 100 setae (Fig. 52). Antennae with 17 articles. Measurements (mm) of 6 imagoes from 1 colony: TBL: 5.23–6.02; HLC: 1.06–1.10; HWE: 1.24–1.27; PL: 0.44–0.65; PW: 0.85–0.98; FWL: 8.02–8.84; ED: 0.27–0.39.

#### Soldier.

(Figs 51–54). Head ellipsoid, covered by about 50 small setae. Labrum elongated, reaching the tip of mandibles, ending in a rounded brush. Postmentum covered by setae from the base to the upper part. Antennae with 13 articles. Mandibles straight, slightly curved at tip, slightly serrated at base. Measurements (mm) of 30 soldiers from 10 colonies: HLC: 1.11–1.33; HLL: 1.51–1.75; HW: 0.95–1.12; PW: 0.53–0.65; RML: 0.66–0.79; MPW: 0.25–0.34; mPW: 0.16–0.22; T3L: 0.74–0.90.

#### Comparisons.

This species is distinguishable from *Parrhinotermes browni* and *Parrhinotermes queenslandicus* Mjöberg, 1920 by the postmentum of soldiers, completely covered by setae.

#### Distribution.

(Fig. 55). This species is common in southern New Guinean forests, and was also collected once in Indonesian Papua.

#### Termitophiles.

The four species of *Parrhinopsenius* found with *Parrhinotermes browni* were also found with this species, previously referred to as *Parrhinotermes* nr. *queenslandicus* (Bourguignon et al. 2007).

#### Etymology.

We named this species after the latin “*barba*”, referring to the postmentum of its soldiers fully covered by setae.

### Genus Schedorhinotermes

Silvestri, 1909

http://species-id.net/wiki/Schedorhinotermes

Rhinotermes (*Schedorhinotermes*) Silvestri 1909: 289.
Schedorhinotermes Silvestri. Snyder, 1949: 89.

#### Type species.

*Rhinotermes intermedius* Brauer, 1865, by original designation.

#### Diagnosis.

Imagoes very similar to those of *Parrhinotermes*. Head approximately circular in shape. Fontanelle situated in the middle of the head. Frons with a slightly visible groove. Labrum short, inclined downward, without groove. Soldiers generally dimorphic and sometimes trimorphic. All species described here have dimorphic soldiers, excepted *Schedorhinotermes seclusus* in which the minor soldiers can sometimes be further separated into two morphs (Miller 1987). Minor soldiers with elongated head. Frons and clypeus with a groove in the middle that joins the opening of the fontanelle to the labrum. Labrum elongated, crossed by a groove in the middle, ending in a brush. Mandibles long and slender. Left mandible with two short subsidiary teeth. Right mandible with one short subsidiary tooth. Major soldiers with labrum proportionally shorter than in minor soldiers. Frons and clypeus with a groove in the middle, from the fontanelle to the labrum. Labrum short and large, with a groove in the middle and an apical brush. Mandibles stout and strongly curved. Left mandible with two large subsidiary teeth. Right mandible with one large subsidiary tooth, as well as a hump at the base. Major soldiers supply more relevant systematic information to distinguish species.

#### Distribution.

This genus is known from Africa, Southeast Asia, the Papuan region and Australia (Emerson 1955, Harris 1968). It feeds on dead wood.

### Schedorhinotermes seclusus

(Hill, 1933)

http://species-id.net/wiki/Schedorhinotermes_seclusus

Figs 56–61 82

Rhinotermes (*Schedorhinotermes*) *intermedius seclusus*Hill 1933: 5.
Schedorhinotermes intermedius seclusus (Hill). Snyder 1949: 92.
Schedorhinotermes seclusus (Hill). Watson et al. 1998: 197.

#### Material examined.

**Lectotype:** **AUSTRALIA: Queensland:** Babinda, 06.i.1925 (G.F. Hill) (NMVA #T-10854). **Other material: PAPUA NEW GUINEA: Southern Highlands:** Lake Kutubu, 13.x.1988 (YR) (#PNGT1295); **Fly:** Morehead, 25.iii.1989 (YR & ML) (#PNGT1438); Morehead, 26.iii.1989 (YR & ML) (#PNGT1453); Wipim, 15.viii.1962 (R. Zweifel), witth nymphs (AMNH); Wipim, 30.iii.1989 (YR & ML) (#PNGT1480, 1482); Wipim, 31.iii.1989 (YR & ML) (#PNGT1495); Wipim, 01.iv.1989 (YR & ML) (#PNGT1504); Lake Murray, 22.v.1990 (YR & ML) (#PNGT1558, 1559, 1560); Lake Murray, 23.v.1990 (YR & ML) (#PNGT1573, 1576); Lake Murray, 24.v.1990 (YR & ML) (#PNGT1579); Lake Murray, 25.v.1990 (YR & ML) (#PNGT1596); Lake Murray, 27.v.1990 (YR & ML) (#PNGT1604); Nomad, 31.v.1990 (YR & ML) (#PNGT1633).

**Figures 56–61.**
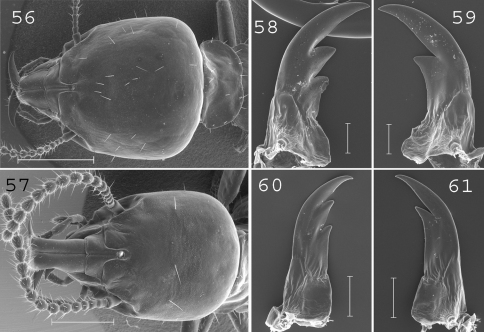
*Schedorhinotermes seclusus*. Major soldier: **56** head; **58** left mandible; **59** right mandible. Minor soldier: **57** head; **60** left mandible; **61** right mandible. Scale bars: **56**, **57**: 0.5mm; **58**, **59**, **60**, **61:** 0.2mm.

#### Imago.

Unknown.

#### Major soldier.

(Figs 56, 58–59) Soldiers of large size. Head covered by about 30 setae. Labrum short and large, not reaching the tip of mandibles. Antennae generally with 17 articles, sometimes with 16 or 18 articles. Pronotum large, covered by about 15 setae principally situated on the edges. Abdomen densely hairy, with 20 to 40 setae per segment. Mandibles moderately curved. Left mandible with the first subsidiary tooth shorter than the second and with a small hump at the base. Right mandible with a slight hump at the base. Measurements (mm) of 1 major soldier from the type colony and 30 major soldiers from 10 colonies (parentheses): HLC: 2.07 (1.82–2.33); HLL: 2.52 (2.24–2.74); HW: 1.96 (1.69–2.05); PW: 1.27 (1.08–1.42); RML: 1.27 (1.00–1.36); mPW: 0.36 (0.22–0.38); T3L: 1.76 (1.43–1.73).

#### Minor soldier.

(Figs 57, 60–61). Soldiers of large size. Head covered by about 15 setae. Labrum 1.5 times longer than wide, reaching the tip of mandibles. Antennae with 16 articles. Pronotum of large size with about 10 setae on the edges. Abdomen abundantly hairy, with 10 to 15 setae per segment. Large mandibles strongly curved. Measurements (mm) of 1 minor soldier from the type colony and 10 minor soldiers from 10 colonies (parentheses): HLC: 1.11 (1.10–1.41); HLL: 1.52 (1.59–1.97); HW: 0.86 (0.95–1.19); PL: 0.44 (0.38–0.48); PW: 0.66 (0.68–0.86); RML: 0.75 (0.79–0.97); MPW: 0.35 (0.34–0.39); T3L: 1.09 (1.01–1.32).

#### Comparisons.

This species is easily distinguishable from others by its large size and its densely hairy abdomen in the minor and major soldier castes. Minor soldiers are highly variable in size, indicating the likely presence of two developmental subcategories (Miller 1987).

#### Distribution.

(Fig. 82). This species is widespread throughout southern Papua New Guinea. It is also known from Queensland, Australia.

#### Termitophiles.

Three species of Trichopseniini (Coleoptera, Staphylinidae, Aleocharinae) were reported by Bourguignon et al. (2007): *Schedolimulus seclusi*, *Schedolimulus planus* and *Papuapsenius magnus*.

### Schedorhinotermes malaccensis

(Holmgren, 1913)

http://species-id.net/wiki/Schedorhinotermes_malaccensis

Figs 62–67 82

Rhinotermes (*Schedorhinotermes*) *malaccensis*Holmgren 1913: 86.
Schedorhinotermes malaccensis (Holmgren). Snyder 1949: 93.

#### Material examined.

**Paratype:** NHRS collections. **Other material: INDONESIA: Papua:** Pusppenssat-IrJa, 13.xi.1995 (YR) (#IRJT15); Road Nabire-Mapia km 48, 15.xi.1995 (YR) (#IRJT43).

#### Imago.

Unknown.

#### Major soldier.

**Figures 62–67.**
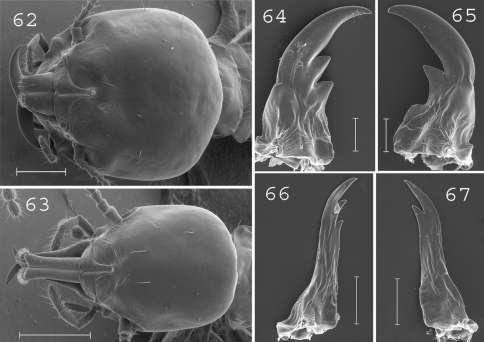
*Schedorhinotermes malaccensis*. Major soldier: **62** head; **64** left mandible; **65** right mandible. Minor soldier: **63** head; **66** left mandible; **67** right mandible. Scale bars: **62**, **63**: 0.5mm; **64**, **65**, **66**, **67:** 0.2mm.

(Figs 62, 64–65). Soldiers of large size. Head rounded, as long as large, covered by about 20 setae. Labrum short and large. Antennae generally with 16 articles. Pronotum large, covered by about 15 setae situated on the edges. Mesonotum and metanotum covered by about 10 setae on posterior margin. Abdomen covered by about 10 setae per segment. Mandibles very large. Right mandible with large hump at the basis. Measurements (mm) of 6 major soldiers from 2 colonies: HLC: 1.70–2.09; HLL: 2.03–2.43; HW: 1.95–2.04; PW: 1.17–1.25; RML: 1.07–1.15; mPW: 0.26–0.34; T3L: 1.54–1.64.

#### Minor soldier.

(Figs 63, 66–67). Head covered by 5 to 10 setae. Labrum moderately elongated, almost reaching the tip of mandibles. Fronto-clypeus short and wide. Antennae with 15 or 16 articles. Pronotum with about 10 setae placed on the edges. Mesonotum and metanotum with about 8 setae on the posterior edge. Mandibles elongated, slender, with short subsidiary teeth. Measurements (mm) of 1 minor soldier from the type colony and 6 minor soldiers from 2 colonies (parentheses): HLC: 1.28 (1.01–1.22); HLL: 1.77 (1.52–1.78); HW: 1.00 (0.81–0.95); PL: 0.50 (0.37–0.49); PW: 0.70 (0.59–0.73); RML: 0.85 (0.72–0.87); MPW: 0.36 (0.30–0.35); T3L: 1.18 (1.03–1.14).

#### Comparisons.

This species is easily distinguished from other New Guinean species by the large rounded head and stout mandibles of its major soldiers.

#### Distribution.

(Fig. 82). This species is widespread throughout Sundaland (Gathorne-Hardy 2004). In New Guinea, it was only collected twice in northwestern Papua.

### Schedorhinotermes longirostris

(Brauer, 1866)

http://species-id.net/wiki/Schedorhinotermes_longirostris

Figs 68–74 82

Termes longirostris Brauer 1866: 47.
Rhinotermes (*Schedorhinotermes*) *longirostris* (Brauer). Holmgren 1913: 86.
Schedorhinotermes longirostris (Brauer). Snyder 1949: 93.
Rhinotermes dimorphus Desneux 1905: 368. syn. n.
Rhinotermes (*Schedorhinotermes*) *dimorphus* Desneux. Holmgren 1911a: 458.
Schedorhinotermes dimorphus (Desneux). Snyder 1949: 90.

#### Material examined.

**Syntypes, minor soldier and workers:** **INDIA: Nicobar Islands:** Kondul Island (NHRS). **Syntypes of *Schedorhinotermes dimorphus*, major soldiers, minor soldiers and workers:** **PAPUA NEW GUINEA: Madang:** Madang (as Friedrich-Wilhelmshafen), 8.i.1901 (L. Biró) (IRSNB). **Other material: PAPUA NEW GUINEA: Sandaun:** Vanimo, 08.iii.1994 (YR & ML) (#PNGT1718); **East Sepik:** Marangis, 07.iii.1983 (JMP & YR) (#PNGT253); **Madang:** Road Madang-Lae km 30, 04.vii.1981 (JMP) (#PNGT120, 121); Bunapas road, 07.vii.1981 (JMP) (#PNGT132, 141); Potsdam plantation, 23.iii.1983 (YR) (#PNGT275); Nubia, 18.v.1983 (YR) (#PNGT353), Potsdam plantation, 20.vi.1983 (YR) (#PNGT386); Bunapae, 25.x.1983 (YR) (#PNGT415); Sepen No.1, 29.x.1983 (YR) (#PNGT425); Potsdam plantation, 25.xi.1983 (YR), with nymphs (#PNGT479); Bunapae, 12.vi.1984 (YR) (#PNGT680); Potsdam plantation, 20.vii.1984 (YR) (#PNGT743); Bunapae, 23.vii.1984 (YR) (#PNGT748); Potsdam plantation, 24.vii.1984 (YR) (#PNGT753); Hansa point, 22.viii.1984 (YR) (#PNGT786); Hansa point, 08.ix.1984 (YR) (#PNGT823); Sepen No.1, 16.ii.1985 (JMP & YR), with alates (#PNGT876); Sepen No.1, 01.iii.1988 (YR) (#PNGT1165, 1166); Yagaum hospital, 10.iv.1983 (YR) (#PNGT304); Gilagil River bridge, 12.iii.1988 (YR) (#PNGT1170); Baitabag, 15.v.1999 (L. Čižek) (#2, J. Šobotník’s collection); Tabobo, 07.i.1989 (ML) (#PNGT1383); Road Kausi-Bundi, 07.v.1988 (YR) (#PNGT1207); Bundi, 10.v.1988 (YR) (#PNGT1210); **Morobe:** Wampit, 06.ii.1983 (JMP & YR) (#PNGT178); Kaiapit, 19.ii.1983 (JMP & YR) (#PNGT192); Sirasira, 15.v.1988 (YR) (#PNGT1219, 1220). Bulolo, 14.ii.1983 (JMP & YR) (#PNGT168); Mount Susu, 23.v.1987 (YR), in branch of klinkii pine on the ground (#PNGT1085); Mount Susu, 19.v.1988 (YR), in branch of klinkii pine on the ground (#PNGT1233); **Oro:** Kokoda, 13.iii.1985 (JMP & YR), in tree stump in cocoa plantation (#PNGT949); Koiasi, 14.iii.1985 (JMP & YR) (#PNGT957); Kokoda, 19.iii.1985 (JMP & YR), in dead wood in rubber plantation (#PNGT987); **East New Britain:** Warengoi, 19.v.1984 (JMP & YR) (#PNGT561); Ataliklikun Bay, 30 km W of Keravat, 23.v.1984 (JMP & YR) (#PNGT595); **New Ireland:** Lelet plateau, 27.v.1984 (JMP & YR) (#PNGT609, 610); Konos, 29.v.1984 (JMP & YR) (#PNGT623); **Manus:** Road Lorengau-Yiringo km 32, 04.vi.1984 (JMP & YR) (#PNGT659); **Central:** Sogeri area, 23.xi.1962 (AE), 2 samples with alates, from logs in rubber grove (AMNH); Sirinumu Dam, 06.iii.1985 (JMP & YR) (#PNGT913); Sirinumu Dam, 08.iii.1985 (JMP & YR) (#PNGT926, 931); **Southern Highlands:** Lake Kutubu, 11.x.1988 (YR) (#PNGT1287, 1288); Pimaga, 16.x.1988 (YR) (#PNGT1304); **Fly:** Tabubil, 20.v.1990 (YR & ML) (#PNGT1548); Lake Murray, 22.v.1990 (YR & ML) (#PNGT1557); Lake Murray, 24.v.1990 (YR & ML) (#PNGT1583); Nomad, 31.v.1990 (YR & ML) (#PNGT1626). **INDONESIA: Papua:** Road Nabire-Mapia km 43, 26.xi.1995 (YR) (#IRJT168); Kaimana, 21.xi.1995 (YR), three samples, the last one with large nymphs (#IRJT103, 109, 113); Kaimana, 23.xi.1995 (YR) (#IRJT142).

#### New synonymy.

We compared the type material of *Schedorhinotermes longirostris* and *Schedorhinotermes dimorphus* with our material and were not able to find any relevant morphological characters to distinguish the two species. Therefore, we consider *Schedorhinotermes dimorphus* as a junior synonym of *Schedorhinotermes longirostris*.

**Figures 68–74.**
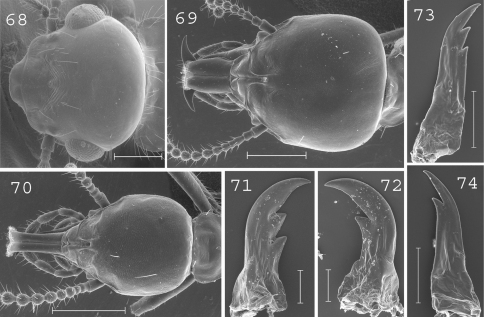
*Schedorhinotermes longirostris*. Imago: **68** head. Major soldier: **69** head; **71** left mandible; **72** right mandible. Minor soldier: **70** head; **73** left mandible; **74** right mandible. Scale bars: **68**, **69**, **70:** 0.5mm; **71**, **72**, **73**, **74:** 0.2mm.

#### Imago.

(Fig. 68). Head covered by 10–15 setae. Eyes of medium size. Pronotum covered by about 50 setae mainly located on the edges. Antennae with 20 articles. Measurements (mm) of 6 imagoes from 1 colony: TBL: 7.39–8.79; HLC: 1.37–1.58; HWE: 1.51–1.62; PL: 0.73–0.79; PW: 1.23–1.36; FWL: 8.70–9.80; ED: 0.31–0.33.

#### Major soldier.

(Figs 69, 71–72). Soldiers of small size. Head, excluding labrum, square-shaped, slightly longer than wide, covered by about 10 setae. Labrum slightly longer than wide, reaching the tip of mandibles. Antennae with 15 or 16 articles. Pronotum covered by 6 long setae disposed in each corners. Mesonotum and metanotum with 4 long setae. Abdomen with 6 long setae per segment, plus sometimes 1 or 2 smaller ones. Mandibles strongly curved at tip with short subsidiary teeth. Left mandible with 2 subsidiary teeth of the same length. Right mandible without hump at the basis. Measurements (mm) of 1 major soldier from the type colony of *Schedorhinotermes longirostris*, 2 major soldiers from the type colony of *Schedorhinotermes dimorphus* [brackets], and 77 major soldiers from 29 colonies (parentheses): HLC: 1.56 [1.50–1.56] (1.35–1.95); HLL: 2.05 [1.92–1.99] (1.65–2.21); HW: 1.32 [1.31–1.35] (1.17–1.51); PW: 0.74 [0.73–0.75] (0.65–0.86); RML: 0.98 [0.89–0.97] (0.75–1.06); mPW: 0.25 [0.24] (0.19–0.30); T3L: 1.19 [1.09–1.16] (1.11–1.36).

#### Minor soldier.

(Figs 70, 73–74). Soldiers of small size. Head elongated, covered by 5–10 setae. Labrum very elongated, 3 times longer than wide, reaching beyond mandibles. Fronto-clypeus of medium size. Antennae with 15 or 16 articles. Pronotum covered by 6 large setae disposed in each corner. Mesonotum and metanotum with 4 long setae. Abdomen with 6 long setae per segment, plus sometimes 1 or 2 smaller ones. Mandibles elongated, hardly curved, with short subsidiary teeth. Measurements (mm) of 10 minor soldiers from 10 colonies of *Schedorhinotermes longirostris*, plus 6 minor soldiers of the type colony of *Schedorhinotermes dimorphus* [brackets]: HLC: 0.82–1.05 [0.90–0.94]; HLL: 1.18–1.54 [1.30–1.37]; HW: 0.60–0.77 [0.66–0.69]; PL: 0.28–0.36 [0.30–0.33]; PW: 0.40–0.53 [0.44–0.51]; RML: 0.52–0.70 [0.51–0.64]; MPW: 0.25–0.31 [0.24–0.28]; T3L: 0.74–0.94 [0.71–0.87].

#### Comparisons.

This species can be distinguished from other New Guinean species by the number of setae on the pronotum (6 per segment), mesonotum (4), metanotum (4) and abdomen (6) in the major soldier caste.

#### Distribution.

(Fig. 82).This species is common in forested areas throughout New Guinea. It probably also occurs in Indonesia, since it was described from the Nicobar islands.

#### Termitophiles.

Several Aleocharinae (Coleoptera, Staphylinidae) were found with this species. *Schedotermoecia kaimanensis* (Coptotermoeciina) was described from colony #IRJT142, and its congener *Schedotermoecia papuana* from #PNGT1165 (Bourguignon and Roisin 2006). The following Trichopseniini also occur with this species: *Schedolimulus elongatus*, *Schedotermoecia planus* and *Schedotermoecia minutides* Bourguignon, *nomen novum* (here proposed as replacement name for *Schedotermoecia minutus* Bourguignon, 2007, to remove secondary homonymy with *Phorilimulus minutus* Pasteels & Kistner, 1971, transferred to *Schedolimulus* by Kanao et al. 2011).

### Schedorhinotermes translucens

(Haviland, 1898)

http://species-id.net/wiki/Schedorhinotermes_translucens

Figs 75–81 83

Termes translucens Haviland 1898: 394.
Rhinotermes translucens (Haviland). Desneux 1904b: 28.
Rhinotermes (*Schedorhinotermes*) *translucens* (Haviland). Holmgren 1911a: 458.
Rhinotermes (*Schedorhinotermes*) *celebensis*Holmgren 1911a: 458. **New synonymy.**
Rhinotermes (*Schedorhinotermes*) *marjoriae*Snyder 1925: 404–405. **New synonymy.**
Schedorhinotermes marjoriae (Snyder). Snyder 1949: 94.
Schedorhinotermes celebensis (Holmgren). Snyder 1949: 90.
Schedorhinotermes translucens (Haviland). Snyder 1949: 96.

#### Material examined.

**Syntypes, all castes:** **MALAYSIA: Sarawak:** Kuching, xi.1894 (G.D. Haviland) (type No. 299, B.M.1899–41, BMNH, collection data from Harris 1966). **Syntype of *Schedorhinotermes celebensis*, alate:** Celebes, Hickson (BMNH). **Other material: SOLOMON ISLANDS:** Guadalcanal, 24.xi.1954 (E.S. Brown), labelled *Schedorhinotermes marjoriae* (BMNH). **PAPUA NEW GUINEA:** **Madang:** Bunapas road, 26.vi.1981 (JMP) (#PNGT104); Nubia, 18.v.1983 (YR) (#PNGT352); Hatzfeldthafen, 20.v.1983 (YR), in bamboo thicket (#PNGT363); Potsdam, 10.xii.1983 (YR) (#PNGT495); Boisa Island, 06.ix.1984 (YR) (#PNGT819); Tabele (Manam Is.), 19.ix.1984 (YR) (#PNGT841); Guam bridge, 12.ii.1985 (JMP & YR) (#PNGT872); Bunapas road, 16.ii.1985 (JMP & YR) (#PNGT877); Hatzfeldthafen, 22.ii.1985 (JMP & YR), with royal pair, 1 alate in log on the ground (#PNGT893); Bogia-Tangu road km 10, 28.vii.1987 (YR) (#PNGT1125 ?1124?); Nubia, 17.ii.1988 (YR), with 3 alates (#PNGT1155); Baitabag, 15.v.1999 (L. Čižek) (#11, J. Šobotník’s collection); Tabobo, 07.i.1989 (ML), with alates (#PNGT1385); Braham mission, 05.v.1988 (YR) (#PNGT1199); **Morobe:** Kaiapit, 18–19.ii.1983 (JMP & YR) (#PNGT185, 190); 19 km W Lae, 28.xi.1962 (AE) (AMNH); 40 km S Lae on Bulolo road, 15.xii.1962 (AE) (AMNH); 21 km ENE Lae, 20.xii.1962 (AE) (AMNH); Markham River (21 km NW Lae), 08.xii.1962 (AE), with nymphoid queen, in standing tree besides stream (AMNH); Oomsis, 25.v.1987 (YR) (#PNGT1089); Bulolo, 14.ii.1983 (JMP & YR) (#PNGT166, 167); Bulolo, 22.v.1987 (YR) (#PNGT1078); 8 km S Bulolo, alt. 900m, 14.xii.1962 (AE) (AMNH); Manki ridge, 18.v.1988 (YR), in *Castanopsis* forest (#PNGT1227); Mount Susu, 19.v.1988 (YR), in hoop pine log (#PNGT1234); Wau-Edie Creek road, 10.ii.1983 (JMP & YR) (#PNGT157, 158); Mount Missim, 12.ii.1983 (JMP & YR) (#PNGT163); Kaulz Creek, 13.xii.1962 (AE), 2 samples from wood stump in mid-montane forest (AMNH); **Eastern Highlands:** Aiyura, 03.i.1963 (AE), 2 samples from stumps, one in *Castanopsis acuminata* forest, one in garden (AMNH); **Sandaun:** Yapsiei, 10.iii.1994 (YR & ML) (#PNGT1731, 1732); Yapsiei, 11.iii.1994 (YR & ML) (#PNGT1743); Yapsiei, 12.iii.1994 (YR & ML), with 1 alate (#PNGT1752); **Manus:** Lorengau-Yiringo road km 32, 04.vi.1984 (JMP & YR) (#PNGT655); Lorengau-Yiringo road km 32, 06.vi.1984 (JMP & YR) (#PNGT674); **East New Britain:** Ataliklikun Bay, 30 km W of Keravat, 23.v.1984 (JMP & YR) (#PNGT596); **Oro:** Kokoda, 13.iii.1985 (JMP & YR) (#PNGT951); Kokoda, 17.iii.1985 (JMP & YR) (#PNGT978, 979); Kokoda, 17.iii.1985 (JMP & YR), in rubber plantation (#PNGT991, 992); **Central:** Sirinumu Dam, 09.iii.1985 (JMP & YR) (#PNGT941); Brown River, 21.iii.1985 (JMP & YR) (#PNGT995); **Southern Highlands:** Bosavi mission, 25.vi.1999 (L. Čižek) (#19, J. Šobotník’s collection); Lake Kutubu, 13.x.1988 (YR) (#PNGT1294); Pimaga, 16–17.x.1988 (YR) (#PNGT1303, 1305, 1311); Pimaga, 19.x.1988 (YR) (#PNGT1320); **Fly:** Morehead, 23.iii.1989 (YR & ML) (#PNGT1417); Wipim, 29.iii.1989 (YR & ML) (#PNGT1474); Tabubil, 19.v.1990 (YR & ML) (#PNGT1538); Lake Murray, 23.v.1990 (YR & ML) (#PNGT1568); Nomad, 29.v.1990 (YR & ML) (#PNGT1615); Nomad, 31.v.1990 (YR & ML) (#PNGT1629); Nomad, 01.vi.1990 (YR & ML) (#PNGT1638, 1641, 1646); Nomad, 02.vi.1990 (YR & ML) (#PNGT1666). **INDONESIA: Papua:** Pusppenssat-IrJa, 14.xi.1995 (YR) (#IRJT25); Road Nabire-Mapia km 48, 15.xi.1995 (YR) (#IRJT42); Road Nabire-Mapia km 62, 18.xi.1995 (YR) (#IRJT69, 70); Pusppenssat-IrJa, 19.xi.1995 (YR) (#IRJT83, 93); Topo, 28.xi.1995 (YR) (#IRJT191); Sanoba, 29.xi.1995 (YR) (#IRJT196); Pusppenssat-IrJa, 01.xii.1995 (YR) (#IRJT212). **Samples included with doubt:** **PAPUA NEW GUINEA: Madang:** Usino, 22.ii.1983 (JMP & YR) (#PNGT215); Nubia, 18.v.1983 (YR) (#PNGT352); Guam bridge, 12.ii.1985 (JMP & YR) (#PNGT868); Hatzfeldthafen, 22.ii.1985 (JMP & YR) (#PNGT892); **East New Britain:** Warengoi, 19.v.1984 (JMP & YR) (#PNGT571).

#### New synonymy.

*Schedorhinotermes celebensis* was described by Holmgren (1911a) based on the alate caste. The distinction of rhinotermitid species based only on alates is uncertain, as only few characters give relevant taxonomic information. After comparison of the type series of *Schedorhinotermes celebensis* and *Schedorhinotermes translucens* it appears that alates of the two species are morphologically identical and could be considered as the same species. Moreover, *Schedorhinotermes celebensis* was mentioned in New Guinea and was therefore expected to occur in our samples. Thus, even though the soldiers could not be compared, we consider *Schedorhinotermes celebensis* as a junior synonym of *Schedorhinotermes translucens*.

*Schedorhinotermes marjoriae* was described by Snyder (1925) based on specimens collected in the Solomon Islands. He pointed out its resemblance with *Schedorhinotermes translucens* and gave as sole character to distinguish these species the morphology of major soldier mandibles. After examination of samples of *Schedorhinotermes marjoriae* and *Schedorhinotermes translucens*, we found that differences between soldier mandibles of the two alleged species are by far smaller than variation observed among New Guinean specimens. For this reason, we also consider *Schedorhinotermes marjoriae* as a junior synonym of *Schedorhinotermes translucens*.

**Figures 75–81.**
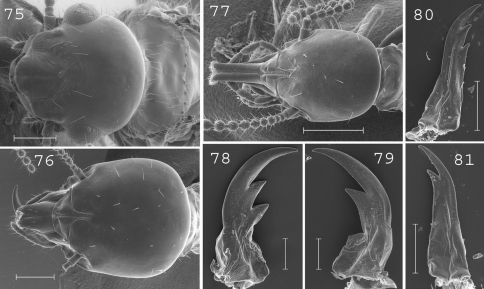
*Schedorhinotermes translucens*. Imago: **75** head. Major soldier: **76** head; **78** left mandible; **79** right mandible. Minor soldier: **77** head; **80** left mandible; **81** right mandible. Scale bars: **75**, **76**, **77:** 0.5mm; **78**, **79**, **80**, **81:** 0.2mm.

**Figure 82.**
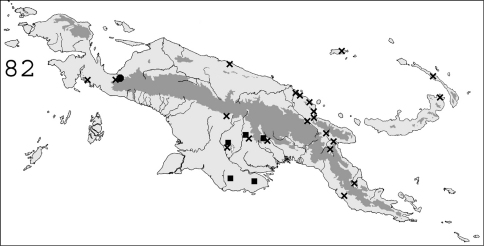
Known distribution in New Guinea of: ■ *Schedorhinotermes seclusus*; ● *Schedorhinotermes malaccensis*; ✖ *Schedorhinotermes longirostris*.

**Figure 83.**
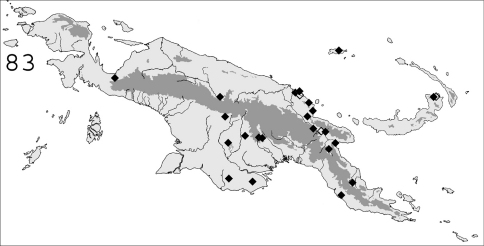
◆ Known distribution in New Guinea of *Schedorhinotermes translucens*; ◇ dubious samples.

#### Imago.

(Fig. 75). Head slightly rounded posteriorly, covered by about 15 setae. Eyes relatively large. Pronotum bearing about 50 setae, principally located on edges. Antennae with 20 articles. Measurements (mm) of 3 imagoes from the type colony of *Schedorhinotermes translucens*, 1 imago from the type colony of *Schedorhinotermes celebensis* [brackets], and 26 imagoes from 5 colonies (parentheses): TBL: 7.31–8.95 [7.18] (7.02–8.95); HLC: 1.62–1.68 [1.54] (1.36–1.67); HWE: 1.72–1.76 [1.66] (1.63–1.84); PL: 0.88–0.98 [0.80] (0.74–0.89); PW: 1.48–1.55 [1.39] (1.30–1.56); FWL: 10.21–10.70 [n.a.] (9.60–11.40); ED: 0.51–0.54 [0.41] (0.31–0.48).

#### Major soldier.

(Figs 76, 78–79). Soldiers of medium size. Head slightly longer than wide, covered by about 30 setae. Labrum not reaching the tip of mandibles. Antennae with 16 articles. Pronotum large, covered by about 15 setae. Mesonotum and metanotum covered by about 10 setae on the posterior margin. Abdomen with 15 to 20 setae per segment. Left mandible with the first subsidiary tooth slightly longer than the second. Right mandible with well developed outgrowth on interior side of base. Measurements (mm) of 3 major soldiers from the type colony of *Schedorhinotermes translucens*, 6 major soldiers from one determined sample of *Schedorhinotermes marjoriae* [brackets], and 87 major soldiers from 29 colonies (parentheses): HLC: 1.91–1.97 [1.95–2.06] (1.45–2.10); HLL: 2.38–2.42 [2.40–2.58] (1.82–2.62); HW: 1.67–1.79 [1.75–1.89] (1.32–1.84); PW: 1.02–1.08 [1.04–1.12] (0.74–1.17); RML: 1.11–1.14 [1.12–1.19] (0.88–1.23); MPW: 0.27–0.30 [0.29–0.34] (0.20–0.34); T3L: 1.54–1.57 [1.42–1.53] (1.15–1.63).

#### Minor soldier.

(Figs 77, 80–81). Head elongated, rounded posteriorly, covered by about 10 setae. Labrum 2.5 times longer than wide, reaching the tip of mandibles. Fronto-clypeus elongated. Antennae with 15 articles. Pronotum with about 10 setae on the edges. Mesonotum and metanotum with about 10 setae on the posterior edge. Abdomen with about 8 to 10 setae per segment. Mandibles slender. Measurements (mm) of 5 minor soldiers from the type colony of *Schedorhinotermes translucens*, 6 minor soldiers of one determined sample of *Schedorhinotermes marjoriae* [brackets] and 10 minor soldiers from 10 colonies (parentheses): HLC: 0.99–1.19 [0.98–1.20] (0.90–1.11); HLL: 1.44–1.72 [1.49–1.67] (1.34–1.68); HW: 0.78–0.93 [0.78–0.87] (0.71–0.90); PL: 0.40–0.48 [0.35–0.46] (0.28–0.43); PW: 0.59–0.69 [0.52–0.63] (0.45–0.65); RML: 0.65–0.78 [0.60–0.72] (0.55–0.77); MPW: 0.30–0.35 [0.28–0.32] (0.24–0.34); T3L: 1.04–1.24 [0.89–1.02] (0.87–1.04).

#### Comparisons.

This species is related to *Schedorhinotermes longirostris*, from which it can be distinguished by its more hairy pronotum, mesonotum, metanotum and abdomen of major soldiers.

#### Distribution.

(Fig. 83). *Schedorhinotermes translucens* is widespread throughout New Guinea, both in savannas and forests. The following additional records are from the literature (samples not examined): Holmgren (1911a): Sattelberg, Kola; as *Schedorhinotermes celebensis*: Aitape (as Eitape (Berlinerhafen)); Roonwal and Maiti (1966): Meervlakte.

#### Termitophiles.

*Myrmedonota termitophila* (Coleoptera, Staphylinidae, Aleocharinae, Lomechusini) was discovered in colony #PNGT163 (Bourguignon and Roisin 2006). The following Trichopseniini (also Aleocharinae), were reported as guests of this species (Bourguignon et al. 2007): *Schedolimulus elongatus*, *Schedolimulus latus*, *Schedolimulus planus*, and *Schizelythron papuanum*.

### Genus Prorhinotermes

Silvestri, 1909

http://species-id.net/wiki/Prorhinotermes

Prorhinotermes Silvestri 1909: 286.

#### Type species.

*Prorhinotermes inopinatus* Silvestri, 1909, by original designation.

#### Diagnosis.

Imago head oval to circular-shaped, with ocelli located before the well developed eyes. Fontanelle situated in the middle of the head. Antenna with 19 to 22 articles. Pronotum narrower than head. Soldier head variable in shape, often larger posteriorly than anteriorly. Fontanelle narrow, placed at anterior third of the head. Frons with a groove in the middle from opening of fontanelle to clypeus. Eyes present as hyaline spots, more or less developed. Antennae with 13 to 20 articles. Pronotum generally wide. Mandibles elongated, left one with a short marginal tooth at the basis, right one without marginal teeth. Soldiers and workers very variable in size (Tho 1992).

#### Distribution.

*Prorhinotermes* has an insular distribution. It is found in the West Indies, Pacific islands, East Indies and islands of the Indian Ocean including Madagascar. It is not reported from continents excepted in Central America, Southern Florida and Northern Australia (Emerson 1952, Gay and Barrett 1983, Roisin et al. 2006).

### Prorhinotermes inopinatus

Silvestri, 1909

http://species-id.net/wiki/Prorhinotermes_inopinatus

Figs 84–87 93

Prorhinotermes inopinatus Silvestri 1909: 287–288.
Prorhinotermes manni Snyder 1925: 399 (synonymized by Snyder 1949: 86).
Prorhinotermes solomonensis Snyder 1925: 400 (synonymized by Snyder 1949: 86).

#### Material examined.

**Syntype:** **TONGA:** Niua Fo’ou (as Insulae Samoa, Ninafoon) (B. Friedländer) (AMNH). **Other material: PAPUA NEW GUINEA:** **Madang:** Bunapas road, 26.vi.1981 (JMP), with neotenic reproductives (#PNGT111); Road Bogia-Tangu km 10, 07.vii.1981 (JMP) (#PNGT129); Potsdam, 18.iv.1983 (YR) (#PNGT325); Nubia, 18.v.1983 (YR) (#PNGT354); Sepen No.1, 29.x.1983 (YR), with nymphs and neotenics (#PNGT426); Bunapae, 12.vi.1984 (YR) (#PNGT687); Bunapae, 23.vii.1984 (YR), with queen in dead wood (#PNGT749); Potsdam, 24.vii.1984 (YR), with neotenics (#PNGT754); Hansa point, 05.ix.1984 (YR) (#PNGT808); Tabele (Manam Is.), 19.ix.1984 (YR) (#PNGT840); Laing Island, 06.ii.1985 (JMP & YR), with alates and neotenics (#PNGT857); Nubia, 25.v.1986 (YR), two colonies, the former dissected completely, with two primary queens and one neotenic male, and nymphs (#PNGT1012, 1013); Potsdam, 03.vi.1986 (YR), two colonies, the latter with many alates (#PNGT1019, 1020); Sepen No.1, 06.vii.1986 (YR) (#PNGT1035); Nubia, 12.vii.1986 (YR), two small colonies (#PNGT1039, 1040); Road Potsdam-Makarup km?, 30.viii.1987 (YR) (#PNGT1137); Sepen No.1, 26.iv.1988 (YR), with two neotenic females (#PNGT1192); Laing Island, 12.iii.1989 (ML), in log on sea shore, with alates (#PNGT1405); Hatzfeldthafen, 20.v.1983 (YR) (#PNGT362); Tabobo, 23.ii.1983 (JMP & YR), with royal pair (#PNGT219); Yagaum Hospital, 07.ii.1983 (JMP & YR), with neotenics (#PNGT149); Yagaum Hospital, 12.iv.1983 (YR), two colonies, the former with alates (#PNGT310, 311); Yagaum Hospital, 13.v.1983 (YR) (#PNGT346); Baitabag, xi.1999 (L. Čižek) (#12, J. Šobotník’s collection). **Morobe:** 21 km NW Lae, 08.xii.1962 (AE), from log on ground in lowland forest (AMNH); Lae, 11.xii.1962 (P. Aloma & AE), rather wet log on forest floor in Botanic Garden (AMNH); 5 km S Lae, 21.xii.1962 (P. Aloma), 2 samples in mangrove branches and stump in water (AMNH); 8 km NW Lae, 27.xii.1962 (AE) (AMNH); 32 km NW Lae (Markham road), 27.xii.1962 (AE), in standing dead tree on edge of *Pandanus* forest (AMNH); 32 km SW Lae, 29.xii.1962 (AE), in sago forest (AMNH); Oomsis, 24.v.1988 (YR) (#PNGT1241); **Sandaun:** Yapsiei, 12.iii.1994 (YR & ML) (#PNGT1753); **Fly:** Tabubil, 20.v.1990 (YR & ML) (#PNGT1539); Nomad, 29.v.1990 (YR & ML), two colonies, the former with neotenics (#PNGT1608, 1612); Nomad, 01.vi.1990 (YR & ML), (#PNGT1635). **INDONESIA:** **Papua:** Pusppenssat-IrJa, 13–15.xi.1995 (YR), three colonies, the first two with neotenics (#IRJT16, 27, 44); Pusppenssat-IrJa, 18.xi.1995 (YR) (#IRJT76); road Nabire-Mapia km 43, 26.xi.1995 (YR) (#IRJT172); Pusppenssat-IrJa, 01.xii.1995 (YR) (#IRJT213). Kaimana, 23.xi.1995 (YR), in dead log on limestone hill (#IRJT145).

#### Imago.

(Fig. 85). Head slightly rounded, covered by about 10 setae. Eyes variable in size. Pronotum with large setae mainly situated on the edges. Measurements (mm) of 12 imagoes from 2 colonies: TBL: 4.38–6.72; HLC: 1.09–1.33; HWE: 1.20–1.47; PL: 0.65–0.81; PW: 1.09–1.27; FWL: 6.67–8.42; ED: 0.24–0.37.

**Figures 84–87.**
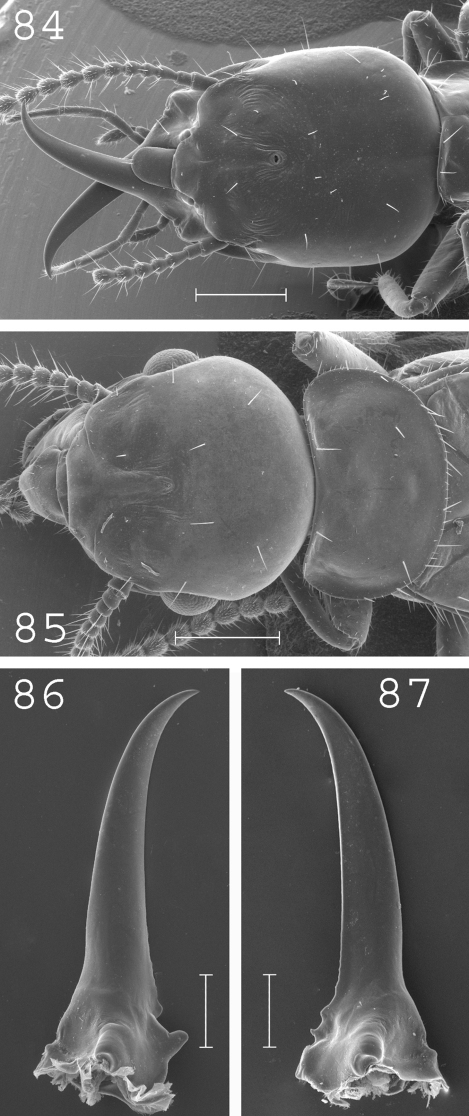
*Prorhinotermes inopinatus.* Soldier: **84** head; **86** left mandible; **87** right mandible. Imago: **85** head and pronotum. Scale bars: **84**, **85:** 0.5mm; **86**, **87:** 0.2mm.

#### Soldier.

(Figs 84, 86–87). Head slightly elongated, covered by about 15 setae. Eyes of medium size. Pronotum, mesonotum and metanotum long and wide. Abdomen covered by 6 to 12 long setae per segment. Large mandibles, slightly curved at tip. Measurements (mm) of syntype and 94 soldiers from 32 colonies (parentheses): HLC: 1.79 (1.39–1.85); HLL: 2.02 (1.65–2.16); HW: 1.10 (1.13–1.53); PL: 0.56 (0.45–0.69); PW: 1.17 (0.86–1.24); RML: 1.22 (0.95–1.40); MPW: 0.51 (0.37–0.57); T3L: 1.35 (1.02–1.48).

#### Distribution.

(Fig. 93). This species occurs throughout New Guinean forests, or forested swamps (Roisin 1988b). It also occurs in Northern Australia (Gay and Barrett 1983), the Solomons, Santa Cruz, Fiji, Samoa, Ellice and Mariana Islands (Hill 1942) and Vanuatu (Roisin et al. 2011).

### Genus Termitogeton

Desneux, 1904

http://species-id.net/wiki/Termitogeton

Termes *(Termitogeton)*Desneux 1904a: 373–374.
Termitogeton Desneux. Holmgren 1911b: 75.

#### Type species.

*Termes planus* Haviland, 1898, by monotypy.

#### Note on type species designation.

Snyder (1949) mentioned *Termes umbilicatus* Hagen, 1858, as type species of *Termitogeton*. However, Desneux (1904a) explicitly based the original description of the subgenus *Termitogeton* on *Termes planus*. In this paper, he mentions *Termes umbilicatus* only once, stating that *Termes planus* “est probablement synonyme de *Termes umbilicatus* Hagen”. That two species are considered probable synonyms by an author does not automatically imply that this author has no doubt about their subgeneric assignment. Consequently, *Termes umbilicatus* should be considered as doubtfully included in *Termitogeton*, and ineligible for type species fixation (Art. 67.2.5 of the Code). *Termitogeton* Desneux should therefore be considered as monotypic when established, *Termes planus* becoming automatically the type species. The fact that Desneux (1904b) subsequently mentioned *Termes umbilicatus* as single valid species of *Termes* (*Termitogeton*), with *Termes planus* as a ?-marked junior synonym, is irrelevant.

#### Diagnosis.

Imagoes densely hairy. Head larger posteriorly than anteriorly. Fontanelle very narrow, placed in the middle of the head. Eyes small. Antennae with 10 to 15 articles. Pronotum very small, half as broad as head. Wings without median and radial vein (Krishna 1970). Soldiers densely hairy, with characteristic heart-shaped, dorso-ventrally flattened head. Antennae generally with 13 to 15 articles. Labrum roughly triangular-shaped. Mandibles elongated, without marginal teeth. Pronotum half as broad as head.

#### Distribution.

*Termitogeton* is a rainforest-dwelling wood feeder known from Sri Lanka, Borneo, Peninsular Malaysia (Tho 1992) and western New Guinea (Parmentier and Roisin 2003).

### Termitogeton planus

(Haviland, 1898)

http://species-id.net/wiki/Termitogeton_planus

Figs 88 93

Termes planus Haviland 1898: 397.
Termes (*Termitogeton*) *planus* Haviland. Desneux 1904a: 373–374.
Termitogeton planus (Haviland). Holmgren 1911b: 75.

#### Material examined.

**Syntypes: MALAYSIA:** **Sarawak:** Santubong, 16.ix.1894 (G.D. Haviland) (type No. 164, CUMZ, collection data from Harris 1966). **Other material:** **INDONESIA: Papua:**Pusppenssat-IrJa (YR): 13.xi.1995 (#IRJT23, in large log on the ground, with alates); 14.xi.1995 (#IRJT26, in large rotten red wood log, with nymphs; #IRJT29); 16.xi.1995 (#IRJT60, small colony with queen); 25.xi.1995 (#IRJT155, 156, 157); 30.xi.1995 (#IRJT202, large colony with alates, nymphs, one neotenic reproductive, in standing dead tree, hard red wood): 01.xii.1995 (#IRJT214, with queen and neotenic reproductives; #IRJT215, 216); Road Nabire-Mapia km 43, 26.xi.1995 (YR) (#IRJT173, 174, in hard red wood). Several of the Indonesian samples mentioned above, then identified as *Termitogeton* nr. *planus*, were previously used in a study of caste patterns (Parmentier & Roisin 2003).

**Figures 88–92.**
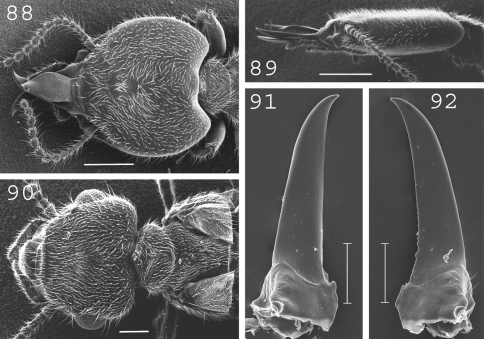
*Termitogeton planus.* Soldier: **88** head and pronotum in dorsal view; **89** head in lateral view; **91** left mandible; **92** right mandible. Imago: **90** head, pro- and mesonotum. Scale bars: **88**, **89:** 0.5mm; **90**, **91**, **92:** 0.2mm.

**Figure 93.**
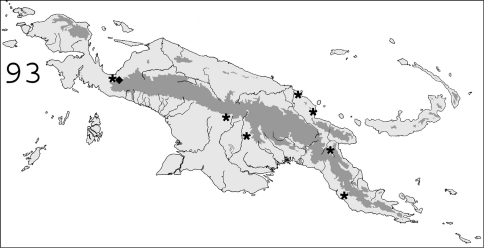
Known distribution in New Guinea of: ∗ *Prorhinotermes inopinatus*; ◆ *Termitogeton planus*.

#### Imago.

(Fig. 90). Very small. Overall body covered by many small setae. Head narrower anteriorly than posteriorly, heart-shaped. Large eyes. Ocelli in front of eyes. Antennae generally with 9 to 15 articles. Pronotum approximately half as broad as head, with a small projection forward. Measurements (mm) of 4 imagoes from the type colony and 10 imagoes from 2 colonies (parentheses): TBL: 3.23–3.94 (3.19–3.94); HLC: 0.59–0.63 (0.59–0.70); HWE: 0.82–0.85 (0.85–0.93); PL: 0.27–0.28 (0.28–0.30); PW: 0.39–0.45 (0.43–0.51); FWL: n.a. (4.93–5.23); ED: 0.14–0.18 (0.18–0.23).

#### Soldier.

(Figs 88–89, 91–92). Overall body covered by many small setae. Head heart-shaped. Labrum elongated, pointed at the tip, narrow at the basis, broadest apical one-third. Antennae with 11 to 15 articles. Pronotum half as broad as head, elongated anteriorly in the middle. Mandibles short, without marginal teeth, slightly curved at tip. Measurements (mm) 4 soldiers from the type colony and 32 soldiers from 8 colonies (parentheses): HLC: 1.16–1.27 (1.10–1.37); HW: 1.21–1.31 (1.12–1.48); PW: 0.51–0.55 (0.45–0.58); LML: 0.71–0.73 (0.75–0.86); PML: 0.87–0.94 (0.77–0.97); mPW: 0.10–0.13 (0.11–0.15); T3L: 0.58–0.62 (0.51–0.65).

#### Distribution.

(Fig. 93).In New Guinea, this species was only collected in the bird’s neck area (western Indonesian Papua). Originally described from from Sarawak (Haviland 1898), it is also known from Sabah (Thapa 1982) and Peninsular Malaysia (Tho 1992).

##### Key to New Guinean Rhinotermitidae species, based on the soldier caste

**Table:** 

1	Mandibles sabre-like, without subsidiary teeth (Figs 14–15)	2
–	Left and right mandibles with 2 and 1 subsidiary teeth respectively (Figs 53–54)	10
2	Head rounded to ovoid, with broad fontanelle opening in front (Fig. 12)	*Coptotermes* 3
–	Head of different shape, with dot-like fontanelle distant from clypeal border	7
3	Head rounded, fontanelle well visible from above, mandibles strongly curved at tip (Figs 6, 7, 9)	*Coptotermes elisae*
–	Soldier head of different shape, fontanelle directed forwards	4
4	Head distincly narrowing anteriorly (Figs 18, 22)	5
–	Head ovoid (Figs 12, 26)	6
5	Small-sized soldier (HW < 1.10 mm), mandibles slightly curved (Figs 22–25)	*Coptotermes pamuae*
–	Large-sized soldier (HW > 1.15) mm, mandibles distinctly curved (Figs 18–21)	*Coptotermes grandiceps*
6	Mandibles curved at tips (Figs 14, 15)	*Coptotermes remotus*
-	Mandibles almost not curved at tips (Figs 28, 29)	*Coptotermes gambrinus*
7	Soldier flattened with heart-shaped head (Fig. 88)	*Termitogeton planus*
–	Soldier not flattened, head ovoid or with parallel sides, with posterior margin not indented (Figs 32, 37, 84)	8
8	Soldier head with sparse setae (Fig. 84)	*Prorhinotermes inopinatus*
–	Soldier head covered by hundreds of setae (Figs 32, 37)	*Heterotermes* 9
9	Head with a small hump anteriorly (Fig. 33); labrum long and pointed, reaching well beyond half length of mandibles	*Heterotermes vagus*
–	Head with a large hump anteriorly (Fig. 38); labrum rounded and short, ending well before half length of mandibles	*Heterotermes paradoxus*
10	Monomorphic soldiers with serrated mandible base (Figs 48, 49)	*Parrhinotermes* 11
-	Polymorphic soldiers, mandible base not serrated	*Schedorhinotermes* 12
11	Postmentum completely covered by setae (Fig. 52)	*Parrhinotermes barbatus*
–	Postmentum covered by setae only in the anterior part (Fig. 47)	*Parrhinotermes browni*
12	Pronotum and abdomen of major soldiers with 6 setae per segment	*Schedorhinotermes longirostris*
–	Pronotum and abdomen of major soldiers with 10 or more setae per segment	13
13	Major soldier head rounded, with stout mandibles (Fig. 62)	*Schedorhinotermes malaccensis*
–	Major soldier head somewhat flattened on sides and posterior margin (Figs 56, 76)	14
14	Abdomen of major soldier with more than 20 setae per segment	*Schedorhinotermes seclusus*
–	Abdomen of major soldier with less than 20 setae per segment	*Schedorhinotermes translucens*

## Discussion

This study reveals that the richness of Rhinotermitidae in New Guinea is much higher than the 8 species previously recognized. Overall, we found 6 genera and 15 species, a diversity which appears slightly lower than that of neighboring areas such as Peninsular Malaysia (6 genera, 24 species (Tho 1992)), Sabah (6 genera, 19 species (Thapa 1982)) and Australia (5 genera, 23 species (Watson and Abbey 1998)). However, these richness figures deserve closer scrutiny, because several species are known only from their original description. Furthermore, the Rhinotermitidae display several characteristics predisposing them to synonymy. (i) In several genera, soldiers may be derived from a series of larval or worker instars, producing a substantial variation in size and number of antennal articles (Hanus et al. 2006). Differences are especially conspicuous between incipient and mature colonies. Therefore, these criteria, though frequently used, are of limited value for species discrimination. (ii) Wood feeders are generally good dispersers and can easily cross salt water gaps by rafting (Eggleton and Tayasu 2001). Extensive colonization of islands and large intercolonial variability favored multiple descriptions of single species under different names along their distribution range. Some pruning, as initiated by Gathorne-Hardy (2004) who placed 11 species of Rhinotermitidae from Sundaland and the western Pacific into synonymy, is clearly needed. On the other hand, the existence of cryptic species has been documented in several Rhinotermitidae (*Heterotermes*, Watson et al. 1989; *Coptotermes*, Brown et al. 1990; *Reticulitermes*, Copren et al. 2005) and Termitidae (*Macrotermes*, Bagine et al. 1994; *Cubitermes*, Roy et al. 2006), so that morphologically homogenous taxa might have to be split when submitted to detailed chemical or molecular analyses.

The distribution patterns of rhinotermitid species match those found in termitids (Roisin 1990, Roisin and Pasteels 1996, 2000, Bourguignon et al. 2008). The central mountain range constitutes a barrier for several species, as in the genus *Parrhinotermes* where *Parrhinotermes browni* occurs almost exclusively on the north slope, while *Parrhinotermes barbatus* occurs in the south. In a similar way, *Heterotermes* is completely missing in the northern part of New Guinea but is represented by two species, also present in Australia, in the south, like the nasute genus *Niuginitermes* (Roisin and Pasteels 1996). Interestingly, northern and southern species meet in the bird’s neck area, where several low valleys connect the two coasts. A second barrier shaping termite species distribution in New Guinea is the ecotone between southern Papuan savannas and the forest-covered areas to the north. Typically, the invertebrate fauna of Papuan savannas shows close affinities with that of northern Australia, whereas forested areas of New Guinea mainly harbor species with Oriental affinities (Gressitt 1982). This situation is illustrated by *Heterotermes vagus* and *Coptotermes pamuae*, which were only found in southern Papuan savannas but also occur in Australia (Hill 1942). Among the Termitidae, at least 11 species (e.g., *Amitermes arboreus*, *Nasutitermes triodiae*, *Microcerotermes taylori*, *Lophotermes aduncus*) are in this case (Roisin 1990, Miller 1994, Roisin and Pasteels 1996, 2000, Bourguignon et al. 2008). Fewer species inhabiting Papuan and northern Australian savannas penetrate deep into southern forests, but *Schedorhinotermes seclusus* and the termitid *Ephelotermes cheeli* do (Bourguignon et al 2008). *Heterotermes paradoxus* was also found in forests in the bird’s neck area. East-west patterns are less conspicuous than north-south ones, but some termitid species show a longitude-restricted range: the genus *Hospitalitermes*, of clear oriental origin, was not found east of Nomad (Roisin and Pasteels 1996), whereas *Microcerotermes piliceps* is restricted to eastern Papua New Guinea and islands further east (Roisin and Pasteels 2000). Here, two species known from as far west as the Malay peninsula, *Schedorhinotermes malaccensis* and *Termitogeton planus*, were recorded only from the bird’s neck area, but their eastern limits are unknown due to the near absence of termite samples from the huge area between the bird’s neck region and the Papua New Guinean border (141° E meridian).

We expect the rhinotermitid diversity found in this study to reflect the overall richness of the island, although it is inevitable that some rare or locally distributed taxa escaped detection. Two regions are particularly likely to host undiscovered taxa: (i) southern Papuan savannas, in which our collecting effort was limited, possibly host additional taxa of Australian affinities; (ii) as stated above, the western half of New Guinea (Indonesian Papua) was also poorly explored and is therefore likely to conceal further taxa of Oriental origin, in the vein of *Termitogeton planus* and *Schedorhinotermes malaccensis*.

## Supplementary Material

XML Treatment for Coptotermes

XML Treatment for Coptotermes elisae

XML Treatment for Coptotermes remotus

XML Treatment for Coptotermes grandiceps

XML Treatment for Coptotermes pamuae

XML Treatment for Coptotermes gambrinus

XML Treatment for Heterotermes

XML Treatment for Heterotermes vagus

XML Treatment for Heterotermes paradoxus

XML Treatment for Parrhinotermes

XML Treatment for Parrhinotermes browni

XML Treatment for Parrhinotermes barbatus

XML Treatment for Schedorhinotermes

XML Treatment for Schedorhinotermes seclusus

XML Treatment for Schedorhinotermes malaccensis

XML Treatment for Schedorhinotermes longirostris

XML Treatment for Schedorhinotermes translucens

XML Treatment for Prorhinotermes

XML Treatment for Prorhinotermes inopinatus

XML Treatment for Termitogeton

XML Treatment for Termitogeton planus

## Appendix 1

**Table 1.** Situation of collecting localities in Papua New Guinea and Indonesian Papua

**Locality **	**S / E**	**Altitude (m a.s.l.)**
Aitape	03°08'S, 142°21'E	
Aiyura	06°20'S, 145°54'E	1700
Ataliklikun Bay	04°20'S, 151°55'E	
Awar	04°08'S, 144°51'E	
Baitabag	05°08'S, 145°46'E	
Bogia-Tangu road km 3-5	04°17'S, 144°57'E	
Bogia-Tangu road km 10	04°19'S, 144°57'E	
Boisa Island	04°00'S, 144°58'E	
Bosavi (mission)	06°25'S, 142°50'E	600
Braham mission	05°45'S, 145°22'E	
Brown River (bridge)	09°14'S, 147°12'E	
Bukaua	06°44'S, 147°22'E	
Bulolo	07°12'S, 146°39'E	800
Bunapae	04°12'S, 144°41'E	
Bunapas road	04°11'S, 144°43'E-50'	
Bundi	05°44'S, 145°14'E	1000
Busu River	06°38'S, 147°01'E	
Coa (Kaimana airfield)	03°39'S, 133°41'E	
Gilagil bridge	04°43'S, 145°39'E	
Guam bridge	04°33'S, 144°58'E	
Hansa Point	04°12'S, 144°55'E	
Hatzfeldthafen (Yoro road)	04°25'S, 145°14'E	
Kaiapit	06°16'S, 146°16'E	
Kaimana	03°39'S, 133°45'E	
Kaulz Creek	07°21'S, 146°46'E	1300
Kausi-Bundi road	05°44'S, 145°18'E	650
Kavieng	02°34'S, 150°48'E	
Koiasi	08°54'S, 147°43'E	
Koil Island	03°21'S, 144°08'E	
Kokoda	08°53'S, 147°44'E	
Kola	05°14'S, 151°25'E	
Konos	03°07'S, 151°43'E	
Lae	06°44'S, 147°00'E	
Laing Island	04°10'S, 144°52'E	
Lake Kutubu (Gesege)	06°27'S, 143°25'E	
Lake Murray	07°02'S, 141°28'E	
Lelet plateau	03°19'S, 151°55'E	950
Lorengau-Yiringo road km 32	02°05'S, 147°09'E	
Madang-Lae road km 30	05°24'S, 145°38'E	
Mambare River	08°03'S, 148°02'E	
Manam Island	04°02'S, 145°00'E04°02'-07', 145°00'-05'	
Manki range	07°15'S, 146°36'E	1350
Marangis	04°01'S, 144°36'E	
Markham River	06°36'S, 146°47'E	
McAdam National Park	07°16'S, 146°38'E	850
Meervlakte	03°20'S, 139°00'E	
Morehead	08°33'S, 141°39'E	
Mount Missim	07°17'S, 146°49'E	1700
Mount Susu	07°14'S, 146°37'E	950
Nabire-Mapia road km 43	03°29'S, 135°40'E	250
Nabire-Mapia road km 48	03°29'S, 135°42'E	350
Nabire-Mapia road km 62	03°31'S, 135°44'E	300
Nomad	06°18'S, 142°14'E	
Nubia	04°11'S, 144°51'E	
Oomsis	06°41'S, 146°48'E	
Pimaga	06°30'S, 143°31'E	800
Popondetta	08°46'S, 148°14'E	
Port Moresby	09°28'S, 147°12'E	
Potsdam (plantation)	04°13'S, 144°56'E	
Pusppenssat-IrJa	03°29'S, 135°43'E	750
Sanoba	03°18'S, 135°34'E	
Sattelberg	06°29'S, 149°49'E	
Sepen No.1	04°11'S, 144°45'E	
Simbang	06°35'S, 147°50'E	
Sirasira	06°19'S, 146°29'E	600
Sirinumu Dam	09°29'S, 147°27'E	
Sogeri	09°25'S, 147°24'E	
Subitana plantation	09°25'S, 147°32'E	
Tabele	04°07'S, 145°00'E	
Tabobo	05°36'S, 145°40'E	
Tabubil	05°17'S, 141°14'E	500
Topo	03°28'S, 135°35'E	
Tsenap	04°16'S, 142°19'E	
UPNG campus	09°25'S, 147°10'E	
Usino	05°34'S, 145°25'E	
Vanimo	02°41'S, 141°18'E	
Varirata National Park	09°26'S, 147°22'E	
Waima	08°38'S, 146°27'E	
Wampit	06°44'S, 146°40'E	
Wanuma	04°54'S, 145°19'E	650
Warengoi	04°31'S, 152°21'E	
Wau-Edie Creek road	07°20'S, 146°40'E	2100
Wipim	08°47'S, 142°52'E	
Yagaum Hospital	05°18'S, 145°45'E	
Yapsiei	04°38'S, 141°06'E	
